# Transcriptome analysis identifies an ASD-Like phenotype in oligodendrocytes and microglia from C58/J amygdala that is dependent on sex and sociability

**DOI:** 10.1186/s12993-024-00240-3

**Published:** 2024-06-19

**Authors:** George D. Dalton, Stephen K. Siecinski, Viktoriya D. Nikolova, Gary P. Cofer, Kathryn J. Hornburg, Yi Qi, G. Allan Johnson, Yong-Hui Jiang, Sheryl S. Moy, Simon G. Gregory

**Affiliations:** 1 Duke Molecular Physiology Institute, Duke University School of Medicine, Durham, NC 27701 USA; 2 Department of Neurology, Duke University School of Medicine, Durham, NC 27710 USA; 3 Department of Psychiatry, The University of North Carolina at Chapel Hill, Chapel Hill, NC 27516 USA; 4 Center for In Vivo Microscopy, Duke University, Durham, NC 27710 USA; 5 Department of Genetics, Neuroscience, and Pediatrics, Yale University School of Medicine, New Haven, CT 06520 USA; 6 Department of Neurology, Molecular Genetics and Microbiology Duke Molecular Physiology Institute, 300 N. Duke Street, DUMC 104775, Durham, NC 27701 USA

**Keywords:** Autism, Oligodendrocyte, Microglia, Myelin, Oxytocin, Amygdala, Brain, Neurodegenerative disease, Glia

## Abstract

**Background:**

Autism Spectrum Disorder (ASD) is a group of neurodevelopmental disorders with higher incidence in males and is characterized by atypical verbal/nonverbal communication, restricted interests that can be accompanied by repetitive behavior, and disturbances in social behavior. This study investigated brain mechanisms that contribute to sociability deficits and sex differences in an ASD animal model.

**Methods:**

Sociability was measured in C58/J and C57BL/6J mice using the 3-chamber social choice test. Bulk RNA-Seq and snRNA-Seq identified transcriptional changes in C58/J and C57BL/6J amygdala within which DMRseq was used to measure differentially methylated regions in amygdala.

**Results:**

C58/J mice displayed divergent social strata in the 3-chamber test. Transcriptional and pathway signatures revealed immune-related biological processes differ between C58/J and C57BL/6J amygdala. Hypermethylated and hypomethylated genes were identified in C58/J versus C57BL/6J amygdala. snRNA-Seq data in C58/J amygdala identified differential transcriptional signatures within oligodendrocytes and microglia characterized by increased ASD risk gene expression and predicted impaired myelination that was dependent on sex and sociability. RNA velocity, gene regulatory network, and cell communication analysis showed diminished oligodendrocyte/microglia differentiation. Findings were verified using Bulk RNA-Seq and demonstrated oxytocin’s beneficial effects on myelin gene expression.

**Limitations:**

Our findings are significant. However, limitations can be noted. The cellular mechanisms linking reduced oligodendrocyte differentiation and reduced myelination to an ASD phenotype in C58/J mice need further investigation. Additional snRNA-Seq and spatial studies would determine if effects in oligodendrocytes/microglia are unique to amygdala or if this occurs in other brain regions. Oxytocin’s effects need further examination to understand its’ potential as an ASD therapeutic.

**Conclusions:**

Our work demonstrates the C58/J mouse model’s utility in evaluating the influence of sex and sociability on the transcriptome in concomitant brain regions involved in ASD. Our single-nucleus transcriptome analysis elucidates potential pathological roles of oligodendrocytes and microglia in ASD. This investigation provides details regarding regulatory features disrupted in these cell types, including transcriptional gene dysregulation, aberrant cell differentiation, altered gene regulatory networks, and changes to key pathways that promote microglia/oligodendrocyte differentiation. Our studies provide insight into interactions between genetic risk and epigenetic processes associated with divergent affiliative behavior and lack of positive sociability.

**Supplementary Information:**

The online version contains supplementary material available at 10.1186/s12993-024-00240-3.

## Background

In 1943, Leo Kanner identified the core symptoms of Autism Spectrum Disorder (ASD) in young children [1]. Today, ASD is considered a highly heterogenous and lifelong neurodevelopmental disorder with onset in infancy or early childhood and diagnostic symptoms that include deficits in social communication and interaction, restricted interests and repetitive behaviors, and sensory anomalies [2]. The incidence of ASD in the United States is estimated at 1 in 36 children with a 4:1 male-to-female ratio [3]. ASD is a group of disorders characterized by multifactor causation with genetic and non-genetic components. Studies have identified over 1,000 genes that could contribute to ASD risk, as well as chromosomal aberrations, genetic syndromes, metabolic disturbances, (mitochondrial dysfunction), epigenetics, and environmental factors [4–6]. Despite the identification of possible underlying mechanisms of ASD, a cohesive model of ASD causation does not currently exist and is a pressing unmet need. Furthermore, behavioral interventions remain the standard of care for ASD with no pharmacological treatments available to address ASD core symptoms. Currently, antipsychotic drugs (e.g., aripirazole, risperidone) are used to alleviate ASD irritability, but these drugs are not consistently effective and have many adverse side effects [7, 8].

There are several potential targeted treatments for ASD including the use of oxytocin which has been explored as a possible ASD therapeutic [9, 10]. Oxytocin is a neuropeptide that is produced in the hypothalamus and secreted both in the blood circulation as a hormone and in the brain where it acts as a neurotransmitter at neuronal oxytocin receptors [11, 12]. In rodents, brain oxytocin receptor expression is both developmentally regulated and sex-specific with males having higher expression in regions such as medial amygdala and the hippocampal CA1 region which mediate core behaviors impaired in autism [13, 14]. Modulation of the oxytocin pathway may be relevant to ASD because the neuropeptide endogenously mediates a range of social behavior. Disruptions of oxytocin signaling has been shown to result in impaired sociability and communication and leads to repetitive behaviors in ASD animal models and humans [12, 15]. In mouse ASD models, oxytocin treatment improved social deficits, social interaction, and social preference [16, 17]. However, clinical studies investigating the efficacy of oxytocin to improve sociability in individuals with ASD, including our own, have yielded mixed results [10, 18]. The failure of oxytocin to act consistently as an ASD therapeutic could be attributed to a lack of understanding about its function. Our current study provides data that helps elucidate the function of oxytocin in a region of the brain that is associated with a core deficit of ASD.

The lack of pharmacological treatments with proven efficacy for ASD is partly because the pathophysiology of ASD remains largely unclear. Abnormal brain growth and structural dissimilarities between ASD and typically developing control brains appear to be important in understanding the symptoms and neuropathology of ASD [19–23]. Alterations in brain morphology in ASD patients are considered by some to be due to brain hypoconnectivity/hyperconnectivity, which leads to imbalances in neuronal excitation/inhibition (E/I) and abnormal brain development and function [24–29]. In support of these hypotheses, studies suggest that ASD anatomical/functional abnormalities are due to myelination deficits, fewer axons, and oxidative stress [27, 30, 31]. However, the specific cellular structures that carry out these processes have still not been adequately identified. Glial cells (oligodendrocytes, astrocytes, microglia) are candidates that may play a role in brain anatomy and functional changes in ASD. They constitute the most abundant cell types in the brain and critically protect the brain’s health under homeostatic conditions. However, in certain pathological states microglia become reactive and secrete proinflammatory cytokines that cause oxidative stress, brain inflammation, neuronal and oligodendrocyte cell death, impaired myelination, neural hypoconnectivity, and dysfunctional synaptic plasticity [32]. Like microglia, astrocytes can also become reactive and release reactive oxygen species (ROS) that damage oligodendrocytes and neurons and cause changes in glutamate transport that results in E/I imbalance that is characteristic of ASD [32]. Thus, reactive glia can contribute to abnormal cytokine profiles in ASD patients as well as altered brain myelination and white matter density. Importantly, alterations in white matter density in brain regions (amygdala, frontal cortex) that play important roles in ASD core symptoms like sociability have been documented in ASD patients [33, 34]. In this study, we attempt to identify the cellular structures that are responsible for ASD pathophysiology as it relates to divergent sociability, and we specifically address the role that glial cells (oligodendrocytes, microglia) and myelination defects play in this process within the amygdala.

Mouse models play an important role in understanding the causes of ASD [35]. C58/J is an inbred mouse strain that exhibits low sociability primarily in males and deficits in social transmission of food preference [36–38]. In the 3-chamber social choice test used to assess sociability, our research group has found that C58/J mice exhibit divergent phenotypes with approximately 50% of mice exhibiting positive sociability and 50% exhibiting social avoidance [37]. C58/J mice also develop motor stereotypic behaviors including backflipping, “jackhammer” jumping, and upright scrabbling which could reflect abnormal repetitive and social behavior in ASD [37, 38]. Studies have demonstrated that oxytocin has prosocial effects in adolescent and adult C58/J mice following a subchronic oxytocin treatment regimen and that enhanced sociability was still present two weeks following treatment [39, 40]. Acute oxytocin also significantly decreased abnormal repetitive behaviors in C58/J mice at doses that did not reduce general locomotion [39].

In the present study, we utilized the C58/J mouse model, with C57BL/6J (B6) serving as a comparison inbred strain, to evaluate the neurological mechanisms that contribute to sociability deficits and sex differences in ASD. C57BL/6J is a highly social strain that appears physically identical to C58/J and that shares genetic lineage with C58/J and thus is an appropriate control for C58/J [41]. The 3-chamber social choice test was used to evaluate sociability in mice. Bulk RNA-Sequencing (Bulk RNA-Seq) and single nucleus RNA-Sequencing (snRNA-Seq) of the amygdala, a brain region altered in individuals with ASD that connects specific neuroanatomical networks that regulate social function, was conducted to correlate transcriptional profiles in this region to strain, sociability, sex, and the effects of oxytocin on behavioral phenotype [42–44]. We interrogated DNA methylation differences in amygdala and analyzed mouse brain connectivity with Magnetic Resonance Histology (MRH). Our analysis of snRNA-Seq data determined that mature oligodendrocytes and microglia exhibit alterations in ASD risk gene expression, genes associated with myelination and microglia homeostatic control, gene regulatory networks, and cell differentiation that are associated with an ASD phenotype in C58/J mice that is dependent on sex and sociability. Bulk RNA-Seq analysis determined that oxytocin treatment had beneficial effects on myelin-related transcriptomic profiles in C58/J amygdala, while immune system-related biological processes that play a role in ASD differed between C58/J and C57BL/6J mice in amygdala. Differences in DNA methylation were also seen between C58/J and C57BL/6J amygdala. This work demonstrates the potential pathological roles of oligodendrocytes and microglia in ASD and provides insight into the mechanisms of oxytocin treatment.

## Methods

### Animals

C57BL/6J and C58/J mice were bred and tested for behavior at the University of North Carolina at Chapel Hill (UNC), with founder breeding pairs obtained from Jackson Laboratories (Bar Harbor, ME). Three separate sets of cohort groups were generated: (1) C57BL/6J and C58/J mice for a between-strain comparison of transcriptional and methylation profiles, (2) C58/J mice to investigate effects of oxytocin treatment on divergent social phenotypes (Supplemental Fig. 1A-D), and (3) C58/J mice for MRH brain analysis. All mice were maintained at 20–23 °C in groups of 2–4 in a specific pathogen-free room on 12 h light and dark cycles with ad libitum access to food and water. All experimental procedures were conducted in compliance with an approved UNC IACUC protocol, and those set forth in the “Guide for the Care and Use of Laboratory Animals” as published by the National Research Council.

### Oxytocin regimen

Oxytocin (Bachem, Torrance, CA) was dissolved in saline containing 0.002% glacial acetic acid. All injections were administered IP (intraperitoneal) in a volume of 10.0 ml/kg. Mice were given 4 injections of vehicle or oxytocin (1.0 mg/kg) over the course of postnatal weeks 6–7 with at least 48 h between each injection. This regimen has previously been shown to have prosocial effects in mice [39, 40].

### Three-chamber choice test

For mice used for the two-strain comparison or for MRI scans, sociability in the 3-chamber test was assessed one time, at age 7–8 weeks, as previously described [45]. In the second set of mice, sociability was assessed 24 h and 2 weeks following the final oxytocin or vehicle treatment, at ages 7–8 and 9–10 weeks. The test started with a 10 min habituation phase, with free exploration of the empty test box. Mice were then given a choice between an unfamiliar stranger mouse contained in a clear plexiglass cage in one side of the test box, or an empty plexiglass cage in the opposite side. Holes were drilled into the cages to allow olfactory investigation of the stranger mouse. Measures were taken of time spent in close proximity (within 5 cm) to the cage containing the stranger mouse or the empty cage by an automated image tracking system (Ethovision, Noldus Information Technology, Wageningen, the Netherlands). To study within-strain divergent social phenotypes, C58/J mice were divided, as evenly as possible, into four groups (male and female low social (ML, FL) and high social (MH, FH)), based on the percent of total proximity time each mouse spent in proximity to the stranger cage. C58/J mice were assigned to “high sociability” and “low sociability” groups by a 50% top/bottom distribution based on our findings on within-strain divergent phenotypes [36]. For control of litter effects in the genetic and epigenetic analyses in this paper, mice were selected starting at the highest and lowest ends of the distribution with the limitation that only one mouse per sex for each sociability group (high or low) could be taken from any one litter with a maximum of three mice per litter. This was done to insure that differences in maternal behavior or other environmental factors did not have a confounding impact on our findings.

### Brain isolation and processing for bulk RNA-Seq

The present study focused on the long-term, persistent transcriptomic and epigenetic changes that could underlie divergent social phenotypes and effects of subchronic oxytocin treatment. In lieu of this focus, perfusion, brain dissection, and tissue collection were performed one day after completion of the 3-chamber choice test in the first set of mice (C57BL/6J and C58/J), and one day after the second 3-chamber test in the second set of mice treated with oxytocin or vehicle (C58/J). The tissue collection was conducted the day after the 3-chamber choice test to control for more transient alterations in gene expression, such as activation of Fos and other immediate-early genes, that occur in acute responses to the 3-chamber choice test itself. The 3-chamber choice test and tissue collection were conducted during the light phase of the light/dark cycle (7:00 am lights on/7:00 pm lights off), with most testing and tissue collection completed between 10:00 am to 2:00 pm. For brain isolation and processing, mice were anesthetized with isoflurane prior to being perfused. Following perfusion, brains were rapidly removed from the skull and were placed into a chilled 1-mm stainless-steel brain matrix and divided into target regions using sterilized razor blades. The amygdala was collected within a 1.2-mm tissue punch. Tissue punches were placed in pre-chilled microcentrifuge tubes on dry ice and were stored at -80 °C.

### Bulk RNA-Seq library preparation, sequencing, alignment, and analysis

For Bulk RNA-Seq analysis in the first set of mice, comparisons were made based on strain, sociability, and sex with C57BL/6J mice (males *n* = 4, females *n* = 4) serving as high-sociability controls, and C58/J mice separated into male and female low sociability (ML, FL, *n* = 4 each) and high sociability (MH, FH, *n* = 4 each) groups. In the second set of mice (all C58/J), comparisons were made based on oxytocin treatment, sociability, and sex with the following groups: ML Vehicle (*n* = 3), ML Oxytocin (*n* = 4), MH Vehicle (*n* = 5), MH Oxytocin (*n* = 4), FL Vehicle (*n* = 4), FL Oxytocin (*n* = 4), FH Vehicle (*n* = 5), and FH Oxytocin (*n* = 4).

DNA and RNA were extracted simultaneously from each sample using the All-Prep DNA/RNA Micro Kit (Qiagen, Hilden, Germany) according to the manufacturer’s instructions. Extracted DNA and RNA samples were quantified on a single-channel spectrophotometer to assess quality by 260/230 and 280/260 absorbance ratios and to determine the approximate yield. RNA samples were stored at -80 °C and DNA was stored at 4 °C. For library preparation, RNA samples were thawed on ice and concentrations were quantified using the Quant-IT™ RiboGreen™ RNA Assay Kit (ThermoFisher, Waltham, MA) and normalized to 10 ng/µL. RNA quality was assessed using high sensitivity RNA ScreenTape analysis run on a 4200 TapeStation (Agilent Technologies, Santa Clara, CA). Libraries were prepared using the TruSeq Stranded mRNA LP kit (Illumina, San Diego, CA) and indexed using the Illumina IDT-TruSeq RNA UD Idx kit. Quality control (QC) of prepared libraries was performed using Agilent high sensitivity D1000 ScreenTape analysis. Completed libraries were transferred to the Duke University Sequencing Core and sequenced on an Illumina NovaSeq 6000 S1 full flow cell. MultiQC reports [46] were generated prior to and after adapter trimming and phredbased read filtering using cutadapt. STAR [47] was used to align reads to the GRCm38 mouse reference genome. The number of reads within each annotated mouse transcript were calculated using FeatureCount [48]. PCAtools package version 2.2 was used to identify outlier samples and to determine factors impacting variation within the expression data. Differential expression analysis was performed using DESeq2 software [49]. To assess differentially expressed genes between C58/J and C57BL/6J amygdala, the design formula for comparisons was strain (as the test variable) + batch + sex + RNA integrity score + 260/230 absorbance ratio + deconvolution-based estimates of L2 IT ENTL, CA3, and oligodendrocyte cell proportions. To assess differentially expressed genes between C58/J mice only, the design formula for comparisons was batch + sex + sociability + treatment + RNA integrity score (with sex, sociability, or treatment as the test variables). For Bulk RNA-Seq analysis of C58/J versus C57BL/6J amygdala, genes with an adjusted *P*-value < 0.05 and Log_2_ Fold Change of > 2 and < -2 were differentially expressed. For Bulk RNA-Seq analysis of C58/J amygdala (MH versus FH, FH Vehicle versus FH oxytocin), genes with an adjusted *P*-value < 0.05 and Log_2_ Fold Change > 0.5 and < -0.5 were considered differentially expressed. For Bulk RNA-Seq analysis of C58/J amygdala (MH versus FH, FH Vehicle versus FH oxytocin), gene-set enrichment analysis (GSEA) was used for biological pathway analysis when comparing C58/J samples and significantly enriched pathways were defined by an adjusted *P*-value < 0.05 and FDR < 0.25 [50]. The clusterProfiler package (version 4.2.2) was used to run GO enrichment pathway analysis of C58/J and C57BL/6J Bulk RNA-Seq data and pathways were considered significant with FDR q-value below 0.05.

### Single nucleus preparation

High quality nuclei were isolated from 10 to 20 mg fresh frozen amygdala tissue pieces using 10x Genomics Chromium Nuclei Isolation Kit (10x Genomics, Pleasanton, CA). Tissue pieces were homogenized by grinding with a pestle and incubated in Lysis Buffer for 10 min on ice. Samples were then dissociated by pipetting, added to a Nuclei Isolation Column, and centrifuged at 16,000 x g for 20 s at 4 °C. The flow through was vortexed, centrifuged, and the nuclear pellet was then washed and centrifuged at 700 x g for 10 min at 4 °C. Nuclear pellets were resuspended in Nuclei Wash and Resuspension buffer. Nuclei were counted and samples were adjusted to 1000 nuclei/µL using a Cellometer K2 cell counter (Nexcelom, Lawrence, MA).

### snRNA-Seq library preparation, sequencing, and analysis

snRNA-Seq libraries were constructed using 10x Genomics Chromium Single Cell 3’ Library & Gel Bead Kit v3 according to the manufacturer’s instructions. Nuclei from 10,000 cells were combined with reverse transcription (RT) reagents and loaded per channel on a 10x Genomics Chromium controller with individually barcoded gel beads and oil to partition each sample into nanoliter-scale gel beads in emulsions (GEMs) within which the RT reaction occurs. GEMs were then broken to reveal full length cDNAs that were purified, amplified, and enzymatically fragmented before Illumina P5 and P7 sequences, sample indexes, and TruSeq Read 2 sequencing primers were added via end repair, A-tailing, adapter ligation, and PCR. cDNAs and final libraries were run on an Agilent 4200 TapeStation to check for quality and were quantified using KAPA Library Quantification Kit (Roche, Basel, Switzerland).

Libraries were transferred to the Duke University Sequencing Core and sequenced using paired end sequencing on an Illumina NovaSeq 6000 S1 full flow cell. Sample demultiplexing, barcode processing, and single-cell 3’ gene counting were performed using 10x Genomics Cell Ranger software. Reads were aligned to the GRCm38 mouse reference genome. The snRNA-Seq data (Cell Ranger result) contained 4 samples from amygdala (ML, MH, FL, FH). The resulting matrix files were used for subsequent bioinformatics analysis in Seurat (version 4.3.0) and R (version 4.1.1). Doublets were removed with the DoubletFinder package (version 2.0.3) and cells with less than 200 genes and more than 5% mitochondria gene count were excluded from analysis due to low quality. Data sets were normalized, logarithmically transformed, and subjected to Principal Component Analysis (PCA). Uniform manifold approximation and projection (UMAP) was utilized to visualize cell clustering. Manual annotation of cell clusters was done by identifying cell types through expression of canonical marker genes [51, 52]. Differentially expressed genes were detected using Seurat’s FindMarkers function. Seurat performs differential expression testing based on the nonparametric Wilcoxon rank sum test. Groups were compared base on sex and sociability and genes with an adjusted *P*-value < 0.05 and Log_2_ Fold Change of > 0.5 and < -0.5 were differentially expressed. The clusterProfiler package (version 4.2.2) was used to run GO enrichment pathway analysis of C58/J snRNA-Seq data and pathways were considered significant with FDR q-value below 0.05. The snRNA-Seq datasets were analyzed with scVelo to determine RNA Velocity [53, 54].

### DNA methylation analysis

Genomic DNA was isolated as described above. DNA (1 ug) was taken to the Duke University Center for Genomic and Computational Biology (GCB) and sheared to achieve 175 bp-long fragments using Covaris S220 (Covaris, Woburn, MA). DNA quality was assessed using Agilent 4200 TapeStation. Libraries were prepared using the Agilent SureSelect^XT^ Methyl-Seq Target Enrichment System. DNA fragments were bead-purified followed by end-repairing and A-tailing. After ligation of methylated adapters, the EZ-DNA Methylation Gold kit (Zymo Research, Irvine, CA) was used to perform bisulfite conversion. After post-bisulfite conversion cleanup, libraries were amplified using PCR and amplicons were purified with AMPure XP beads (Beckman Couter, Brea CA) and indexed with Agilent SureSelect^XT^ Index Set ILM. Quality control of libraries was done using Agilent high sensitivity D1000 ScreenTape and TapeStation analysis. Libraries were then sequenced on an Illumina NovaSeq S1 full flow-cell. Reads from the DNA methylation libraries were processed and mapped to GRCm38 using Bismark version 0.22.3 [55] and bowtie2 version 2.4.1 [56]. CpG methylation values were extracted from the aligned bisulfite converted genomes using the Bismarck methylation extractor function. The R package Rnbeads version 2.8 [57] was used to quality assess the DNA methylation datasets and identify differential methylation at individual CpG resolution. Methylation coverage files generated by the Bismarck methylation extractor were annotated via the Rnbeads pipeline using the GRCm38 reference genome. Differentially methylated regions were determined using the R package dmrseq [58] and the BSmooth R package [59]. CpGs were considered differentially methylated at an FDR (q score) > 0.1. For the strain comparisons in the methylation analysis, the design formula for comparisons was strain (test variable) + batch + sex + extraction yield (ng/µL) + deconvolution-based estimates of L2 IT ENTL, CA3, and oligodendrocyte cell proportions. Since genetic variation between C57BL/6J and C58/J mice had the potential to interfere with the sequence-specific baits utilized by the SureSelect methylation kit, spot checks were performed on differentially methylated regions identified by DMRseq and Rnbead platforms to determine if divergent features of the respective strains could potentially skew the results.

### SCENIC analysis

The R packages SCENIC (version 1.1.2-01), RcisTarget (version 1.20.0), and AUCell (version 1.22.0) were used to identify transcription factors and cell states in snRNA-Seq data from C58/J amygdala [60]. Raw UMI counts from Seurat served as input matrices for each sample in SCENIC. A gene filter was applied that kept genes with at least 6 UMI counts across all samples that were detected in at least 1% of the cells. GENIE3 (version 1.22.0) was used to identify potential transcription factor targets. The activity of each regulon was evaluated using AUCell which calculates the area under the recovery curve and integrates the expression ranks across all genes in a regulon. Cells were clustered according to gene regulatory network or regulon activity.

### Cell-To-cell communication analysis

The R package CellChat (version 1.6.1) was used to analyze intercellular communications within the snRNA-Seq datasets from C58/J amygdala [61]. CellChat is a public database of ligands, receptors, cofactors, and their interactions. The CellChat R toolkit and a Web-based “CellChat Explorer” (http://www.cellchat.org/) were used to identify intercellular communication and help construct cell-cell communication atlases. For the cell-interaction analyses, the expression levels were calculated relative to the total read mapping to the same set of coding genes in all transcriptomes. The expression values were averaged within each single-cell cluster or cell sample.

### Magnetic resonance histology

C58/J mice underwent transcardial perfusions with a mixture of 10% buffered formalin and Prohance (Gadoteridol) to reduce the spin lattice relaxation time and enhance the signal for MRH [62]. Images were acquired on a 7 T horizontal bore magnet with Agilent Direct Drive console and Resonance Research high performance gradients with peak gradients of 2000 mT/m. The head of the perfused specimen with the brain in the cranial vault was placed in a 12 mm diameter solenoid radiofrequency coil. Diffusion tensor MR images were acquired using a Stesjkal Tanner spin echo imaging sequence with TR/TE = 100/15.8 ms with diffusion weighting (bvalue) of 3000 s/mm^2^. Forty-six diffusion weighted images were acquired using gradient vectors equally distributed on the unit sphere. Baseline (b_0_) images were acquired after every tenth volume. The acquisitions were accelerated using compressed sensing with a compression factor of 8 resulting in isotropic resolution of 35 μm with acquisition time of 22 h. Volumes were registered together, denoised and processed using a series of imaging pipelines described fully in [63]. Image volumes were registered to a standardized atlas with a label set consistent with the ABA CCFv3 [63]. This results in a collection of 1800 regions of interest (ROI) in each hemisphere. Diffusion tensor images for each specimen were mapped into a common space so we could compare the volume of each ROI and the mean scalar metrics for each ROI. These scalar measures of diffusion properties included the axial diffusivity (AD), mean diffusivity (MD), radial diffusivity (RD), and fractional anisotropy (FA) which are sensitive to changes in tissue cytoarchitecture (axonal density, axon size and distribution, myelin degradation). Statistical comparisons of volume and scalar diffusion properties of each of the 360 ROI between groups (ML, MH, FL, FH) were performed in Matlab using a Kruskal Wallis nonparametric Anova [64]. Connectomes were also generated between all of the ROI using DSI Studio [63, 65]. The connectome measures the strength of connectivity between any given pair of ROI derived from a tracking algorithm. An Omni-MANOVA analysis described more fully in [66] was used to reduce the dimensionality of the comparison and determine regional differences in connectivity between groups. Composite image were created for each group using the QSDR method [67].

## Results

### C58/J mice display divergent levels of sociability and differentially expressed genes associated with ASD compared to C57BL/6J mice

**Fig. 1:**
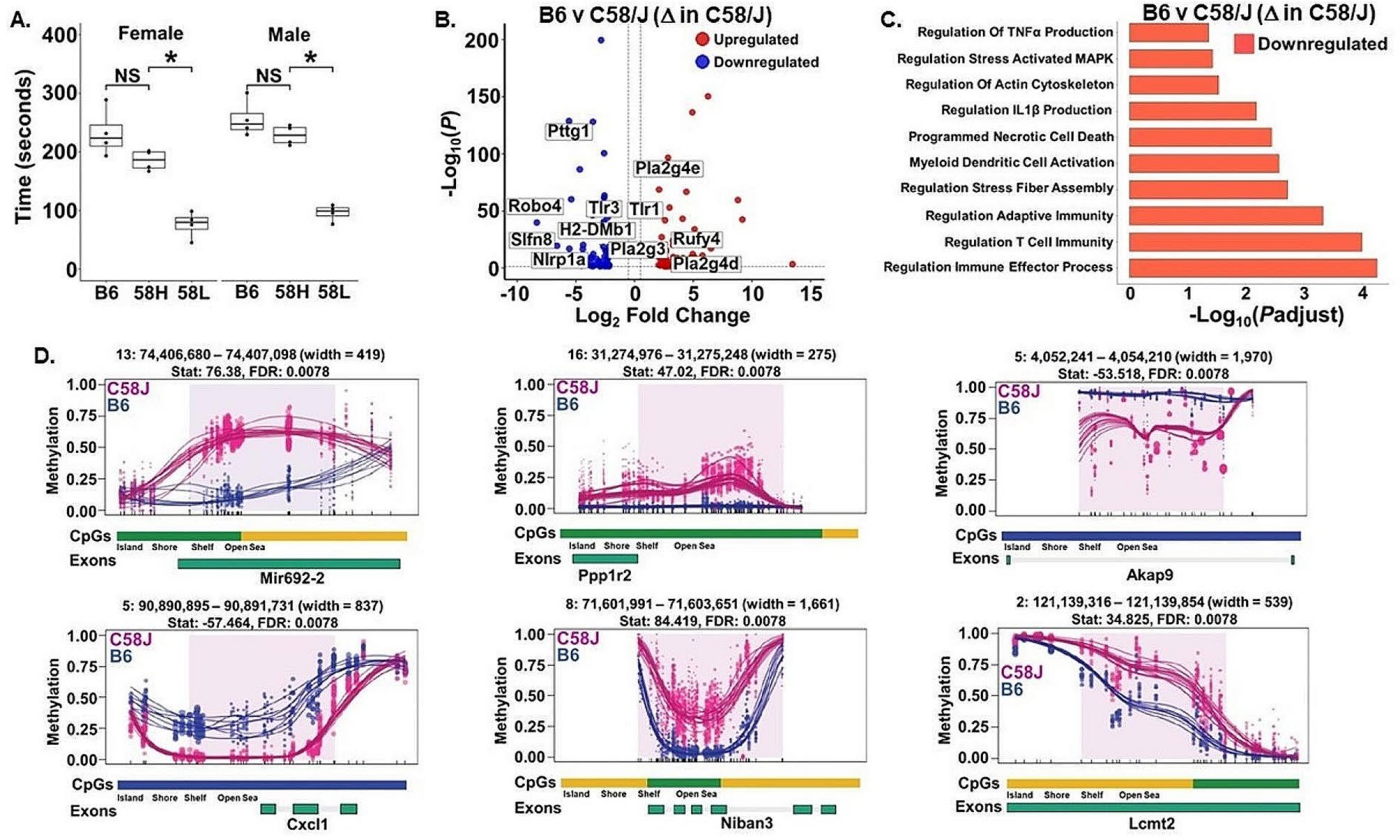
C58/J mice exhibit reduced sociability and downregulation of immune-related pathways in amygdala compared to C57BL/6J mice. (**A**) Comparison of social preference based on time spent in close proximity to a stranger mouse during the 3-chamber social choice test in male (*n* = 4 per group) and female (*n* = 4 per group) C57BL/6J (B6), C58/J high social (58 H), and C58/J low social (58 L) mice. Preference for social interaction was assessed at postnatal weeks 7–8. Results reported as mean ± SEM, *p < 0.05 using Mann-Whitney U test. (**B**) Volcano plot of differentially expressed genes obtained from bulk RNA-Seq analysis of amygdala from C58/J and C57BL/6J mice (*n* = 4 mice per group). Genes with an adjusted *P*-value < 0.05 and Log_2_ Fold Change of > 2 and < -2 were differentially expressed. 64 genes were upregulated and 63 genes were downregulated in C58/J. (**C**) GO enrichment analysis of Bulk RNA-Seq data from C58/J and C57BL/6J amygdala. Pathways shown were significant (FDR *q*-value < 0.05) and were downregulated in C58/J mice. (**D**) Significant (*q*-value < 0.05) hypermethylated (*Mir692-2*, *Niban3*, *Ppp1r2*, *Lcmt2*) and hypomethylated (*Cxcl1*, *Akap9*) regions identified in C58/J compared to C57BL/6J mice (*n* = 4 mice per group). Lines represent individual smoothed methylation level estimates for C58/J (C58J, red) or C57BL/6J (B6, blue). Dots represent methylation level estimates of an individual CpG in a single sample, and dot size is representative of coverage. CpG and genic annotation tracks are shown below each plot

Our study replicated previous findings of high and low sociability phenotypes within the isogenetic C58/J inbred strain [45]. Notably, the high sociability C58/J mice spent similar amounts of time in proximity to the stranger mouse as the C57BL/6J controls, while the low sociability groups had significantly lower duration than both the high-sociability C58/J mice and C57BL/6J (Fig. 1A, see also Supplemental Fig. 1). Bulk RNA-Seq differential gene expression analysis was conducted in the amygdala to gain insight into the molecular mechanisms that contribute to strain-level differences in sociability between C58/J and C57BL/6J mice. With an adjusted p-value < 0.05 and log_2_-FoldChange of 2 and − 2, the Bulk RNA-Seq results identified 64 upregulated and 63 downregulated genes in C58/J amygdala (Fig. 1B).

Many of the differentially expressed genes (DEGs) identified were associated with immune function and evidence highlights a link between immune dysfunction and ASD [68]. For instance, elevated levels of the enzyme Phospholipase A2 (Pla2) are associated with ASD, while *Pla2* genes have been shown to be differentially expressed in ASD [69, 70]. *Pla2g4d* and *Pla2g4e* were upregulated in C58/J amygdala, while *Pla2g3* was downregulated in comparison to C57BL/6J (Fig. 1B, Supplemental Table 1). Studies also suggest that innate-immunity-related pathways play an important role in autism and have identified several genes in these pathways that are differentially expressed in ASD [71]. Multiple DEGs in C58/J amygdala encode proteins that are part of innate-immunity-related pathways including *Treml2* (myeloid cell innate immunity), *Pycard* (Nod-like receptor signaling), *Tlr3*/*Tlr1* (Toll-like receptor signaling), and *Ccl21a* (Chemokine signaling) (Fig. 1B, Supplemental Table 1).

Like innate immunity, neuronal migration plays an important role in the developing brain and is often impaired in ASD. Recent studies have shown that neuronal migration is reduced in mouse brain when members of the brain complement pathway, such as Masp1 and Masp2, are decreased and our findings show *Masp2* gene expression is increased in C58/J amygdala (Supplemental Table 1) [72]. Genetic analyses indicate the axon guidance molecule *Robo4* may play a role in ASD by affecting serotonin signaling or neurodevelopment and *Robo4* was overtly downregulated in C58/J amygdala (Fig. 1B, Supplemental Table 1) [73]. Finally, autophagy is a degradation mechanism that helps maintain neuronal homeostasis, survival, and plays a role in the progression of neuronal disease. The *Rufy4* gene that encodes a protein responsible for macroautophagy was highly upregulated in C58/J amygdala (Fig. 1B, Supplemental Table 1) [74]. The analysis of enriched Gene Ontology (GO) signaling pathways revealed many of the downregulated genes were enriched in immune-related biological processes such as regulation of TNFα and IL1β production, regulation of T Cell immunity, programmed necrotic cell death, regulation of adaptive immunity, and myeloid dendritic cell activation (Fig. 1C).

In addition to alterations in gene expression and signaling pathways, epigenetic mechanisms may shed light on what distinguishes C58/J mice from C57BL/6J mice. Differential methylation analysis conducted using DMRseq between C58/J and C57BL/6J identified 248 differentially methylated regions (DMRs) in amygdala (100 hypomethylated, 148 hypermethylated). The DMRs in amygdala overlapped with 70 annotated genes. The top hypermethylated genes in C58/J compared to C57BL/6J amygdala overlapped with the following genes at *q*-value = 0.5 × 10^− 2^: the micro-RNA *Mir692-2*, Apoptosis Regulator 3 (*Niban3*), Protein Phosphatase 1 Regulatory Inhibitor Subunit 2 (*Ppp1r2*), and Leucine Carboxyl Methyltransferase 2 (*Lcmt2*) (Fig. 1D). Two of the top hypomethylated genes in C58/J relative to C57BL/6J amygdala were C-X-C Motif Chemokine Ligand 1 (*Cxcl1*) and A-Kinase Anchoring Protein 9 (*Akap9*) (Fig. 1D). Several members of the A-kinase anchor protein (Akap) family, including Akap9, are functionally and genetically linked to ASD, while studies have shown levels of the immunological marker Cxcl1 were significantly elevated in ASD patients [75, 76]. In addition to the aforementioaned genes, Rnbeads analysis also identified 55 CpG islands and 274 promoters that were differentially methylated between C58/J and C57BL/6J mice (data not shown).

In addition to attempting to uncover the molecular mechanisms that differentiate C58/J mice from C57BL/6J mice, we utilized MRH to examine variations of brain architecture in C58/J mice as a function of sex and sociability (Supplemental Fig. 2). MRH analysis revealed no differences in whole-brain connectomes or in regional brain volumes between ML, FL, MH, and FH mice suggesting that divergent sociability could not be attributed to overt neuroanatomical or connectivity differences.

### C58/J mouse amygdala consists of 29 cell types with distinct ASD-like transcriptomic signatures that were dependent on sex and sociability

**Fig. 2:**
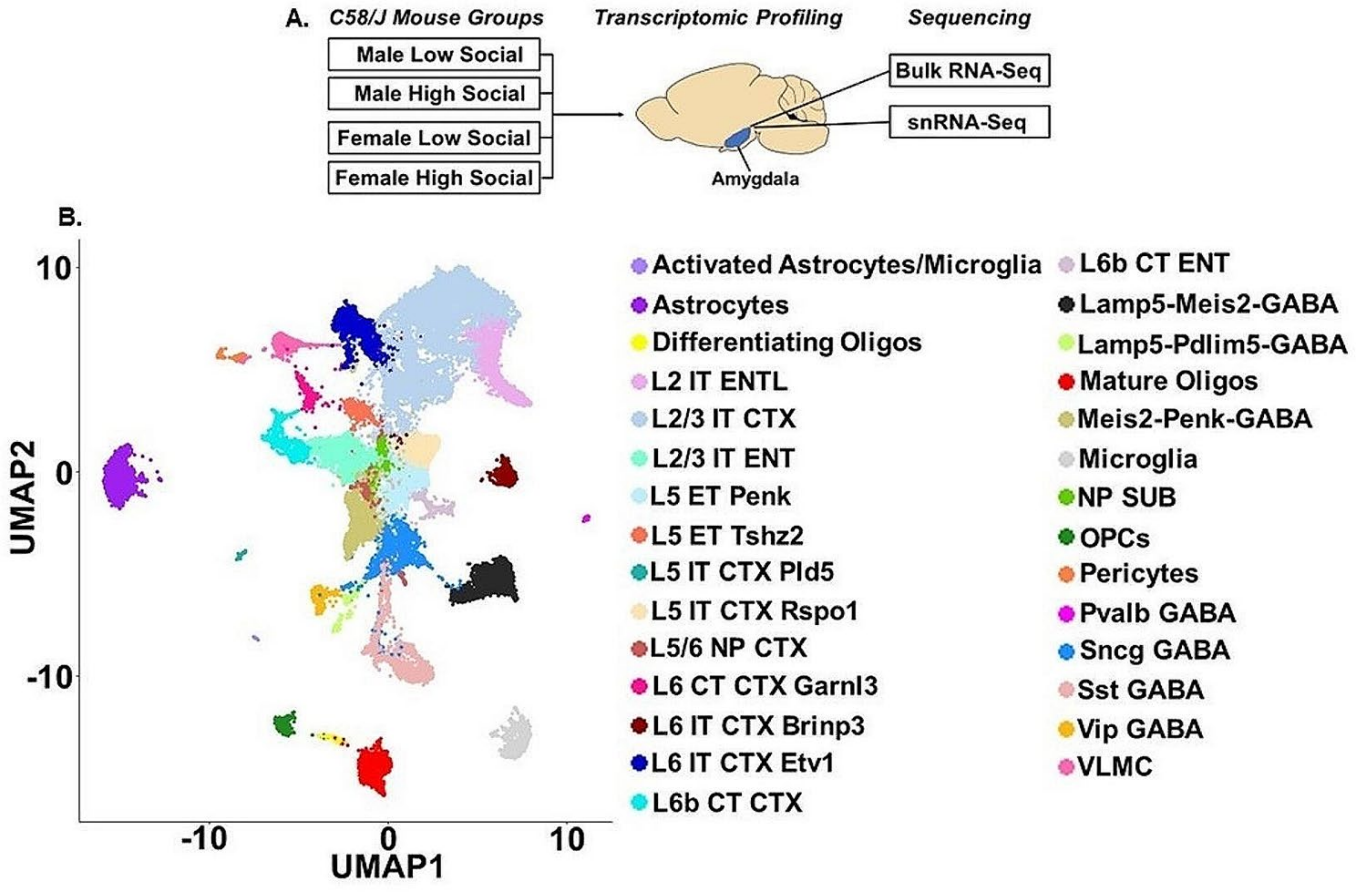
Clustering of 31,447 cells identified 29 cell types in C58/J amygdala. (**A**) Amygdala was dissected from brains taken from C58/J Male Low Social (ML), Male High Social (MH), Female Low Social (FL), and Female High Social (FH) mice (*n* = 1 mouse per group) and was used for Bulk RNA-Sequencing (Bulk RNA-Seq) and single nucleus RNA-Sequencing (snRNA-Seq). (**B**) UMAP plot of 31,447 cells and the 29 distinct cell clusters that were obtained from snRNA-Seq

Mice were sacrificed and amygdala were collected for Bulk RNA-Seq and snRNA-Seq analysis (Fig. 2A). A quality-controlled single cell atlas of C58/J amygdala included 31,447 cells from ML, MH, FL, and FH mice (*n* = 1 mouse per group). Unsupervised Seurat analysis revealed 29 distinct UMAP clusters (Fig. 2B), which were annotated using canonical markers of the mouse cortex from previous studies (Table 1) [51, 52]. These 29 cell populations could be simplified into 3 main cell types: glutamatergic (*Slc17a7*/*Slc17a6*, *Celf2*, *Arpp21*, *Pcsk2*, *Ptprd*, *Ano3*) (Supplemental Fig. 3A-B), GABAergic (*Adarb2*+/*Adarb2*-, *Gad1*, *Gad2*, *Grip1*, *Dlx6os1*) (Supplemental Fig. 4A-B), and non-neuronal (Supplemental Fig. 5). Based on the enriched gene features, the four C58/J groups showed notable variability of cell type proportions. For instance, the ML data contained elevated numbers of L2/3 IT CTX cells (40.3%, 2981 cells), while MH had a greater number of Meis2-Penk-GABA cells (13.5%, 963 cells) and the FH had increased L5 IT CTX Rspo1 cells (12.9%, 936 cells) (Supplemental Fig. 6A-B). In addition, the MH and FL mice had a greater number of astrocytes (9.3%, 699 cells; 8.6%, 820 cells), microglia (5.6%, 400 cells; 5.0%, 475 cells) and mature oligodendrocytes (6.3%, 450 cells; 7.1%, 675 cells) compared to the ML (astrocytes 2.75%, 201 cells; mature oligodendrocytes 0.66%, 49 cells; microglia 1.0%, 74 cells) and FH (astrocytes 5.1%, 368 cells; mature oligodendrocytes 1.0%, 74 cells; microglia 1.2%, 89 cells) (Supplemental Fig. 6A).

**Table 1:** Cell types and their gene signatures in C58/J amygdala

Cell Type	Cell Type Gene Signature
L2/3 IT CTX	*Cux2*, *Rtn4rl1*, *Slc30a3*, *Lrrtm4*
L2 IT ENTL	*Grik1*, *Pdzrn3*, *Plcxd3*, *Efna5*
L6 IT CTX Brinp3	*C1ql3*, *Cdh13*, *Brinp3*
L5 ET Tshz2	*Tshz2*, *Sgcz*, *Tox*
L6b CT CTX	*Ccn2*, *Hs3st4*, *Cdh18*, *Vwc2l*
L6b CT ENT	*Tfap2d*, *Zfpm2*, *Tshz2*
L5/6 NP CTX	*Grik1*, *Mgat4c Tshz2, Kcnip1*
L5 IT CTX Pld5	*Car10*, *Pld5*, *Hs6st3*, *Kctd8*
L5 IT CTX Rspo1	*Car10*, *Prr16*, *Brinp3*, *Zbtb7c*, *Rspo1*
NP SUB	*Foxp2*, *Kcnip1, Tshz2, Grik1*
L5 ET Penk	*Sgcz*, *Penk*, *Rarb, Tshz2*
L2/3 IT ENT	*Lrrtm4, Car10*
L6 CT CTX Garnl3	*Garnl3*, *Grp*, *Syt6, Hs3st4*
Lamp5-Pdlim5-GABA	*Lamp5*, *Pdlim5*, *Nxph1*, *Ndnf*
Lamp5-Meis2-GABA	*Lamp5*, *Meis2*, *Pbx3*, *Chst9*
Sncg GABA	*Ntng1*, *Pcdh11x*, *Pax6*, *Luzp2, Sncg*
Vip GABA	*Vip*, *Tac2*, *Crh*, *Cnr1*
Pvalb GABA	*Pvalb*, *Lrrc4c*, *Thsd7a*, *Cntnap5c*, *Kcnmb2*
Sst GABA	*Sst*, *Sox6*, *Grin3a*, *Grm1*
Meis2-Penk-GABA	*Meis2*, *Penk*, *Sp8*, *Lockd*
OPCs	*Pcdh15*, *Pdgfra*, *Lhfpl3*
Differentiating Oligodendrocytes	*Itpr2*, *Bcas1*, *Tnr*
Mature Oligodendrocytes	*Trf*, *Mal*, *Prr5l*
Astrocytes	*Slc1a2*, *Plpp3*, *Wdr17*
Microglia	*Selplg*, *Hexb*, *Siglech*
Pericytes	*Rgs5*, *Kcnj8*, *Abcc9*
VLMC	*Col3a1*, *Bnc2*, *Col25a1*
Activated Astrocytes/Microglia	*Csf1r*, *Trem2*, *Itgb2*, *Mertk*, *Bcl6*, *Fcgr1*

Next, we stratified our snRNA-Seq and Bulk RNA-Seq data based on sex (ML vs. FL, MH vs. FH) and sociability (ML vs. MH, FL vs. FH) given the core ASD epidemiological features of social dysfunction and a greater frequency in males. Bulk RNA-Seq comparison between MH vs. FH showed the greatest differences in gene expression (239 genes) compared to the other groups (ML vs. FL 10 genes, ML vs. MH 45 genes, FL vs. FH 12 genes) (Supplemental Figs. 7&8). These 239 genes included seven high confidence (HC) and strong candidate (SC) ASD risk genes from the Simons Foundation Autism Research Initiative (SFARI) ASD risk gene database (Supplemental Fig. 8B). Pathway analysis of the Bulk RNA-Seq DEGs revealed that many of the top enriched Gene Ontology (GO) terms in the MH vs. FH comparison could be functionally related to ASD pathogenesis, including synapse organization, learning, protein demethylation, axonogenesis, and synaptic plasticity (Supplemental Fig. 9A).

**Fig. 3:**
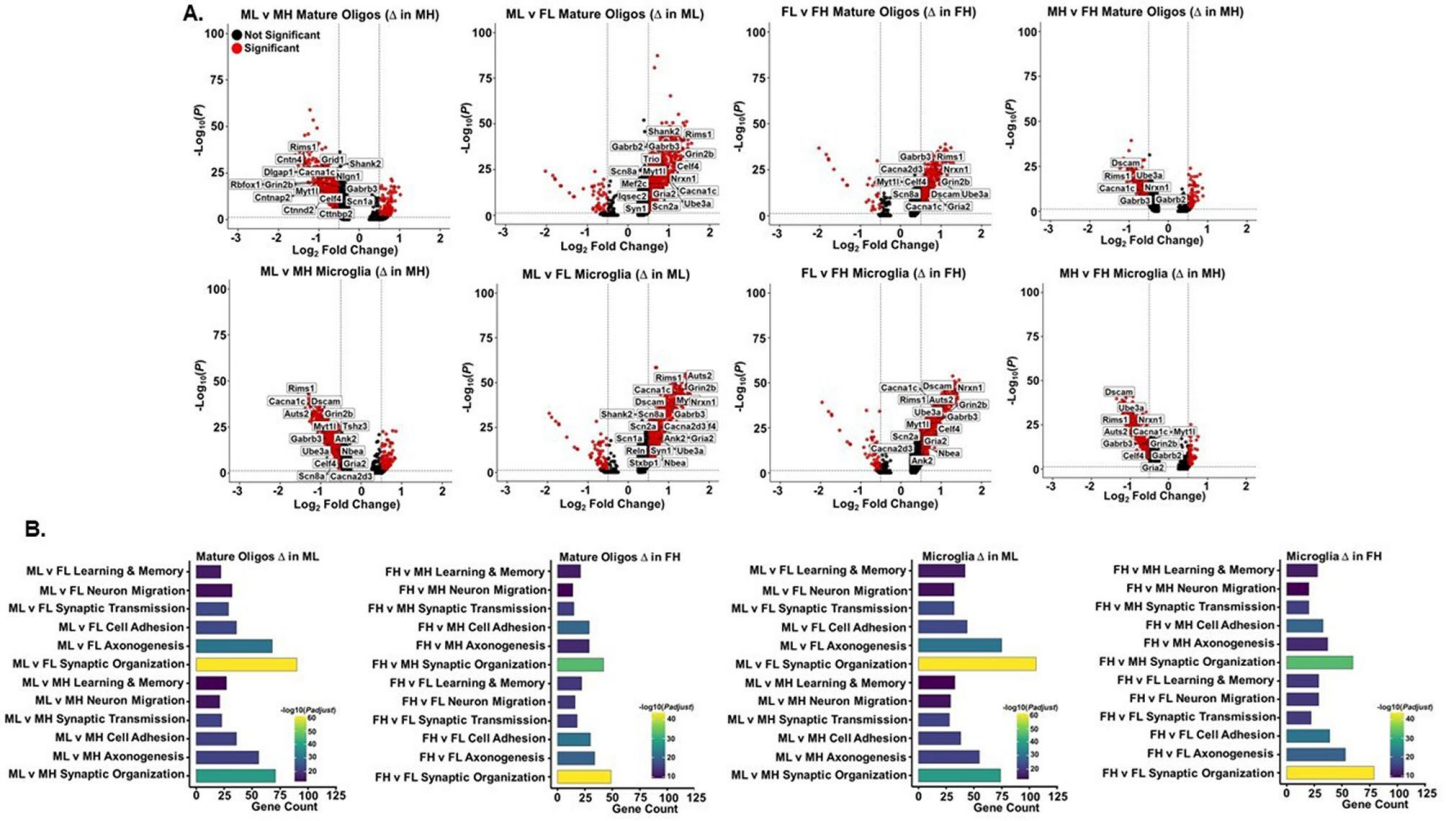
Mature oligodendrocytes and microglia from Male Low Social (ML) and Female High Social (FH) C58/J amygdala demonstrate the greatest number of differentially expressed SFARI ASD risk genes. (**A**) Volcano plots showing differentially expressed genes (DEGs) obtained in microglia and mature oligodendrocytes when C58/J mouse groups (*n* = 1 mouse per group) were compared based on sex and sociability. Specific high confidence (HC) SFARI ASD risk DEGs are labeled in the plots. (**B**) Gene Ontology Biological Process (GO BP) enrichment analysis of ASD-related pathways in mature oligodendrocytes and microglia when C58/J mouse groups were compared based on sex and sociability. Pathways shown were significant (FDR *q*-value < 0.05) and were upregulated in ML and FH. Female Low Social, FL; Male High Social, MH

Analysis of the snRNA-Seq data revealed DEGs and SFARI risk genes within ML and FH cell types (Supplemental Figs. 7&8). Interestingly, mature oligodendrocytes and microglia contained the greatest number of DEGs across the four comparisons (Supplemental Figs. 7&8). Compared to the FL and MH groups, ML and FH mature oligodendrocytes and microglia showed the greatest differences in gene expression (Fig. 3A). GO term analysis of the snRNA-Seq dataset revealed that many ASD-related pathways were enriched specifically in microglia and mature oligodendrocytes with the greatest number of DEGs and SFARI risk genes occurring in the ML group when compared to MH (sociability) and FL (sex) (Fig. 3A, B).

### Dysregulation of myelin-related gene expression and myelin-related pathways in mature oligodendrocytes from ML and FH C58/J amygdala

**Fig. 4:**
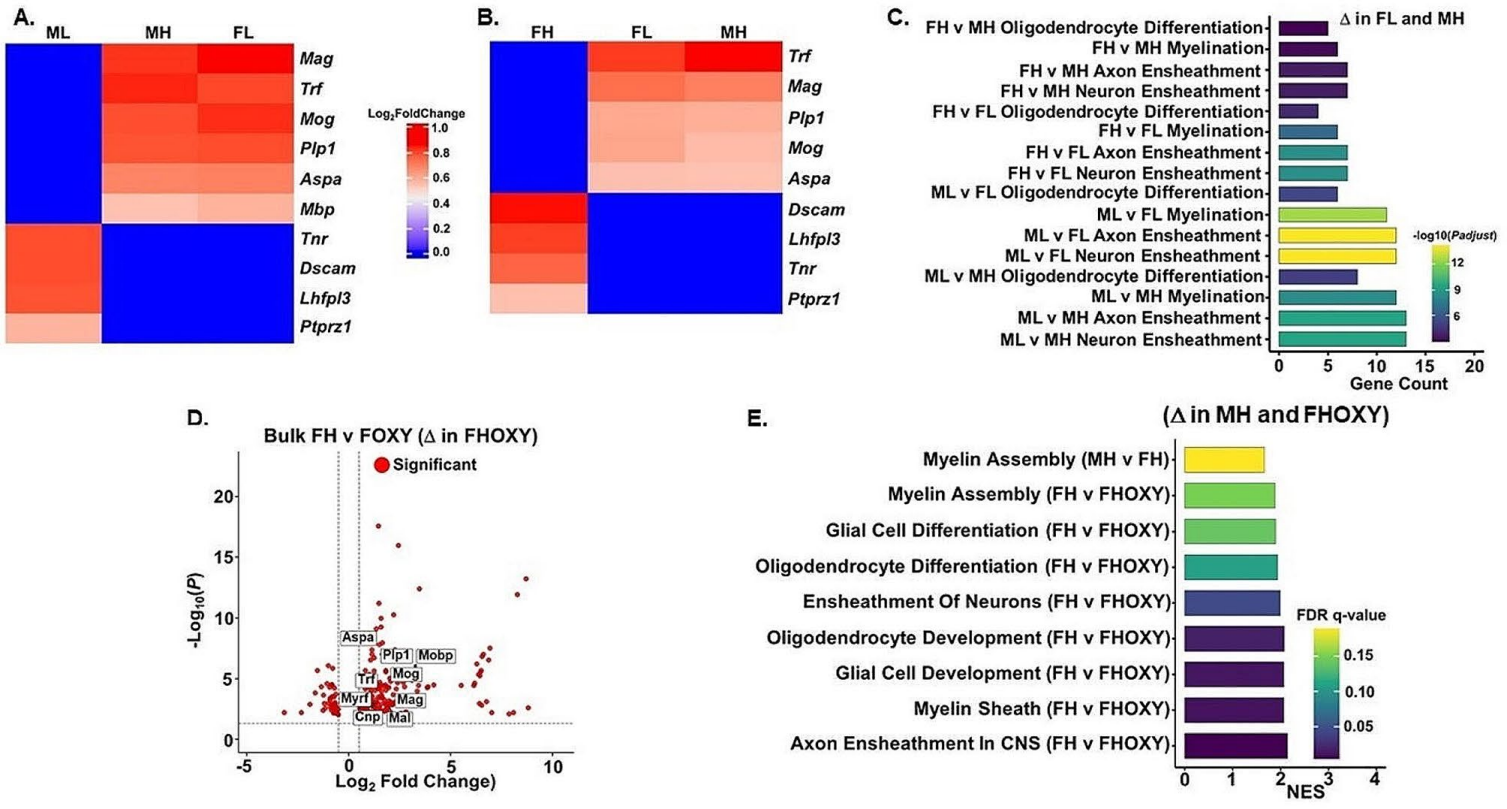
Dysregulation of myelinating gene expression and myelinating pathways in mature oligodendrocytes from Male Low Social (ML) and Female High Social (FH) C58/J amygdala. (**A**) Heatmap of genes in ML mature oligodendrocytes of C58/J amygdala (*n* = 1 mouse per group) that are differentially expressed compared to Male High Social (MH) and Female Low Social (FL) mature oligodendrocytes. Genes are markers for mature oligodendrocytes (*Aspa*, *Mog*, *Mag*, *Plp1, Trf, Mbp*) and OPCs (*Ptprz1*, *Tnr*, *Dscam*, *Lhfpl3)*. Genes with an adjusted *P*-value < 0.05 and Log_2_ Fold Change of > 0.5 and < -0.5 were differentially expressed. (**B**) Heatmap of genes in FH mature oligodendrocytes of C58/J amygdala (*n* = 1 mouse per group) that are differentially expressed compared to FL and MH mature oligodendrocytes. Genes are markers for mature oligodendrocytes (*Aspa*, *Mog*, *Mag*, *Plp1, Trf, Mbp*) and OPCs (*Ptprz1*, *Tnr*, *Dscam*, *Lhfpl3)*. Genes with an adjusted *P*-value < 0.05 and Log_2_ Fold Change of > 0.5 and < -0.5 were differentially expressed. (**C**) Gene Ontology Biological Process (GO BP) enrichment analysis of myelination-related pathways in mature oligodendrocytes from representative groups of C58/J amygdala that were compared based on sex and sociability. Pathways shown were significant at an FDR *q*-value < 0.05 and were upregulated in MH and FL. (**D**) Volcano plot showing differentially expressed genes (DEGs) obtained by Bulk RNA-Seq in Female High Social amygdala vehicle-treated (FH) and oxytocin-treated (FHOXY) groups (*n* = 4–5 mice per group). Specific myelinating genes are labeled in the plot. Genes are differentially expressed in the FHOXY group. Differentially expressed genes were defined by a log2FC > 0.5 and *P*adjust value < 0.05. (**E**) GO BP enrichment analysis of pathways involved in myelination in Male High Social (MH) amygdala compared to Female High Social (FH) amygdala and FH vehicle-treated amygdala compared to FH oxytocin-treated (FHOXY) amygdala obtained by Bulk RNA-Seq analysis and GSEA (*n* = 4–5 mice per group). Positive normalized enrichment score (NES) indicates pathways are upregulated in MH and FHOXY groups. The significantly enriched pathways were defined by nominal *P* value < 0.05 and FDR < 0.25

Next, we focused on the interactions between sex and sociability on oligodendrocyte expression given the differences in mature oligodendrocyte numbers in ML and FH together with the differential expression of ASD risk genes in this cell type. MH and FL oligodendrocytes were enriched in myelin-related genes, such as *Aspa*, *Mag*, *Mog*, and *Plp1*, which are characteristic of mature differentiated myelinating oligodendrocytes (Fig. 4A, B) [77]. GO pathway analysis showed enrichment in biological processes related to myelination, including neuron/axon ensheathment, oligodendrocyte differentiation, and myelination, in MH and FL (Fig. 4C). In contrast, ML and FH oligodendrocytes had elevated expression of genes like *Ptprz1*, *Tnr*, *Dscam*, and *Lhfpl3* that indicate an OPC-like phenotype (Fig. 4A, B). Bulk RNA-Seq analysis by sex and sociability corroborated our findings related to myelination.

To further investigate expression profiles associated with sociability, analyses were carried out for a separate set of C58/J mice given a subchronic regimen with either oxytocin or vehicle. We found that oxytocin treatment did not have significant effects in comparisons between transcription in high sociability and low sociability male C58/J mice. However, in female mice, the high sociability group treated with oxytocin (FHOXY) exhibited upregulation of genes involved in myelin synthesis (*Trf*, *Aspa*, *Plp1*, *Mog*, *Mal*, *Mobp*, *Myrf*, *Cnp*, *Mag*) in amygdala compared to vehicle-treated FH mice (Fig. 4D). Signaling pathways related to myelination were also upregulated in amygdala from FHOXY compared to vehicle-treated FH, as well as MH amygdala compared to FH amygdala (Fig. 4E).

**Fig. 5:**
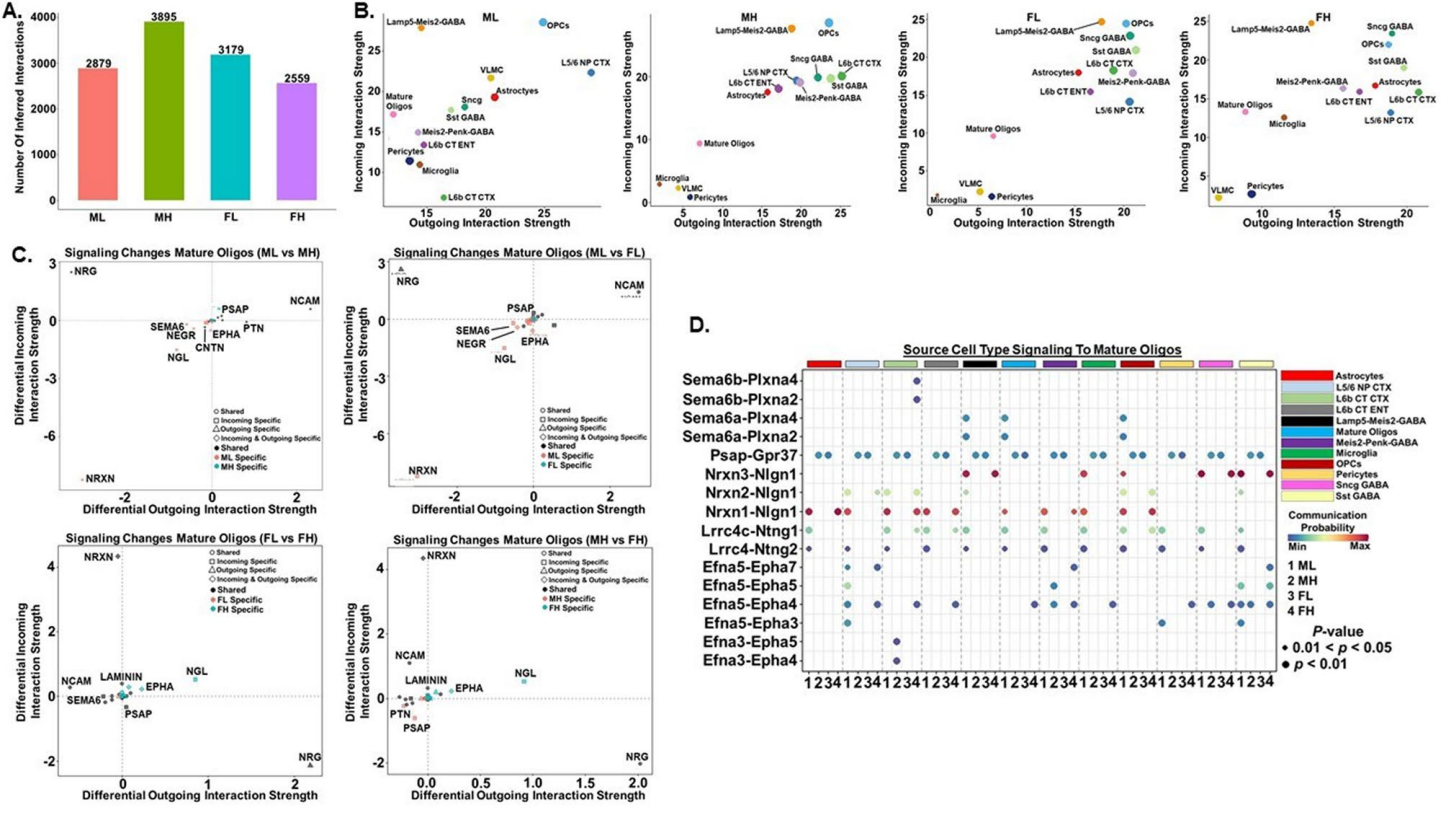
Intercellular communication networks that are important in mature oligodendrocytes in C58/J amygdala. (**A**) Total number of inferred ligand-receptor interactions in Male Low (ML), Male High (MH), Female Low (FL), and Female High (FH) amygdala. (**B**) Scatter plot of incoming and outgoing interaction strength in ML, MH, FL, and FH groups. (**C**) Signaling changes in mature oligodendrocytes in MH (compared to ML), FH (compared to FL), FL (compared to ML), and FH (compared to MH) (**D**) Bubble plots of the communication probability of the ligand-receptor interactions that contribute to signaling from the indicated cell types to mature oligodendrocytes. Ligands originate from the indicated 12 source cell types and interact with receptors in mature oligodendrocytes from representative groups of C58/J amygdala (*n* = 1 mouse per group). Color implies communication probability and dot size indicates specificity of the interaction

Cell-cell communication plays an important role in coordinating processes such as cellular differentiation and tissue homeostasis. Importantly intercellular communication networks can differ between healthy and diseased models. We utilized the R package CellChat to quantitatively determine intercellular communication networks from snRNA-Seq data generated in amygdala of male and female low and high social C58/J mice [61]. CellChat analysis revealed 2879 total ligand-receptor interactions in the ML group, 3895 ligand-receptor interactions in the MH group, 3179 ligand-receptor interactions in the FL group, and 2559 ligand-receptor interactions in the FH group (Fig. 5A). Ligand-receptor interaction strength is quantified by a probability value that is modeled by the law of mass action which is based on the ligand average expression value in a specific cell type and the average expression value of a receptor on another cell type, along with cofactors of the ligands and receptors [61]. The total interaction strength of the ML (221) group was moderately higher than that of the MH (203), FL (191), and FH (194) groups (data not shown). When the outgoing and incoming signals of various cell populations were analyzed in the ML, MH, FL, and FH groups, we found that the mature oligodendrocytes were a signaling source in all four samples (Fig. 5B). However, mature oligodendrocytes in the ML and FH were a more prominent signaling target compared to the MH and FL (Fig. 5B).

We next investigated the changes in individual signaling pathways in the mature oligodendrocytes in each of the four samples. Compared to MH and FL, ML mature oligodendrocytes exhibited increased incoming and outgoing neurexin (NRXN), netrin (NGL), semaphorin 6 (SEMA6), and neuronal growth regulator (NEGR) signaling (Fig. 5C). The ML mature oligodendrocytes also had increased incoming ephrin (EPHA) signaling compared to MH and FL (Fig. 5C). These changes in NRXN, NGL, SEMA6, NEGR, and EPHA signaling were ML-specific meaning signaling from these pathways was undetectable in the MH and FL mature oligodendrocytes (Fig. 5C). Similarly, increased prosaposin (PSAP) signaling was specific to MH and FL compared to ML mature oligodendrocytes (Fig. 5C). Like the ML, FH mature oligodendrocytes exhibited specific increases in EPHA and NGL incoming and outgoing signaling compared to MH and FL mature oligodendrocytes, while incoming NRXN signaling was increased (Fig. 5C). The MH and FL mature oligodendrocytes also exhibited increased PSAP signaling compared to FH mature oligodendrocytes (Fig. 5C).

Finally, we examined the specific ligand-receptor pairs in the SEMA6, PSAP, NGL, NRXN, and EPHA pathways utilized by different cell types to communicate with mature oligodendrocytes in the four samples. Multiple NRXN ligand-receptor pairs (NRNX3-NLGN1, NRXN1-NLGN1) were the dominant signaling molecules utilized by various cell types to signal to the ML and FH mature oligodendrocytes (Fig. 5D). Neurexins and neuroligins (NLGN) are cell adhesion molecules that regulate synaptogenesis and synaptic transmission and alterations in neurexin/neuroligin genes are associated with ASD [78, 79]. The Netrin-G ligand LRRC4C is a synaptic adhesion molecule that interacts with the presynaptic adhesion molecule netrin-G1 (NTNG1) and the ligand-receptor pair LRRC4C/NTNG1 has been implicated in ASD [80, 81]. The LRRC4C/NTNG1 ligand-receptor pair were also prominent NGL signaling molecules used by multiple cell types to signal to ML mature oligodendrocytes (Fig. 5D).

Ephrin receptors and their ligands also comprise an important signaling class in neurons that play a role in axon guidance, synapse formation, and synaptic plasticity [82]. Studies suggest ephrin ligand-receptor pairs also regulate signaling between axons and oligodendrocytes that precedes axon myelination [83]. Multiple EPHA ligand-receptor pairs were utilized by different cell types to signal exclusively to ML and FH mature oligodendrocytes (Fig. 5D). Semaphorins are involved in many processes in the nervous system where they bind to plexin (Plxn) receptors to produce their effects [84]. SEMA6A regulates the timing of oligodendrocyte differentiation and myelination which was confirmed in SEMA6A-knockout mice in which oligodendrocyte differentiation is delayed [85]. SEMA6A ligand-receptor pairs (SEMA6A-PLXNA4, SEMA6A-PLXNA2) were increased in multiple ML cell types that signal to mature oligodendrocytes (Fig. 5D). SEMA6B is thought to compensate for the loss of SEMA6A in SEMA6A-KO mice and SEMA6B ligand-receptor pairs (SEMA6B-PLXNA2, SEMA6B-PLXNA4) were upregulated in FH L6B CT CTX neurons that signalled to FH mature oligodendrocytes (Fig. 5D) [85, 86]. Finally, GPR37 is highly expressed in the nervous system where it controls myelination by regulating the differentiation of oligodendrocytes into mature myelinating oligodendrocytes [87]. Studies have also shown that GPR37-knockout mice exhibit reduced expression of the myelin associated protein MAG and myelination [88]. An increase in PSAP-GPR37 ligand-receptor pairs was seen in all MH and FL cell types that signaled to mature oligodendrocytes compared to ML and FH samples (Fig. 5D).

### Homeostatic gene expression and cell-cell communication are altered in microglia from ML and FH C58/J amygdala

**Fig. 6:**
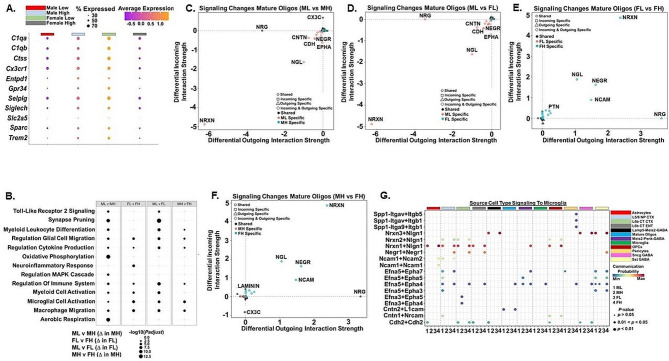
Homeostatic gene expression and cell-cell communication are altered in microglia from Male Low Social (ML) and Female High Social (FH) C58/J amygdala. (**A**) Dot plot of genes in microglia from representative groups of C58/J amygdala (*n* = 1 mouse per group) that are markers for microglial homeostasis. Color intensity implies level of gene expression and dot size indicates the percentage of each cluster expressing the gene. (**B**) Dot plots of GO Biological Process (GO BP) enrichment analysis of immune/microglial-related pathways in microglia from representative groups of C58/J amygdala that were compared based on sex and sociability (*n* = 1 mouse per group). Dot sizes are proportional to –log10(*P*adjust). Pathways presented were considered significant with FDR q-value below 0.05. Female Low Social, FL; Male High Social, MH. (**C-F**) Signaling changes in microglia in MH (compared to ML), FL (compared to ML), FH (compared to FL), and FH (compared to MH). (**G**) Bubble plots of the communication probability of the ligand-receptor interactions that contribute to signaling from the indicated cell types to microglia. Ligands originate from the indicated 12 source cell types and interact with receptors in microglia from representative groups of C58/J amygdala (*n* = 1 mouse per group). Color implies communication probability and dot size indicates specificity of the interaction

Like mature oligodendrocytes, reductions in cell density and upregulation of ASD risk genes were observed in ML and FH microglia (Fig. 3A and Supplemental Fig. 6A). Microglia in ML and FH exhibited reduced levels of homeostatic genes such as *C1qa*, *C1qb*, *Ctss*, *Cx3cr1*, *Entpd1*, *Gpr34*, *Selplg*, *Siglech*, *Slc2a5*, *Sparc*, and *Trem2* (Fig. 6A). GO pathway analysis revealed oxidative phosphorylation (OXPHOS), aerobic respiration, regulation of the immune system, and synaptic pruning were elevated in MH microglia compared to ML microglia suggesting microglia were more quiescent in MH compared to ML (Fig. 6B). Synaptic pruning and several immune-related pathways were also elevated in FL microglia compared to ML microglia (Fig. 6B). Interestingly, pathways related to the neuroinflammatory response and regulation of the immune system were elevated in FL microglia compared to FH microglia (Fig. 6B). Pathway analysis of Bulk RNA-Seq data supported our snRNA-Seq data that differences were observed in immune system-related pathways and OXPHOS between MH and FH amygdala (Supplemental Fig. 9B-D).

We utilized CellChat to analyze the intercellular communication networks between different cell types and microglia in amygdala. When the outgoing and incoming signals of various cell populations were analyzed in the ML, MH, FL, and FH groups, we found that the microglia were not a significant signaling source or target in MH and FL amygdala (Fig. 5B). In contrast, microglia in the ML and FH were a signaling source and target (Fig. 5B). We then analyzed the changes in individual signaling pathways in the microglia in each of the four samples. Compared to MH and FL, ML microglia exhibited increased incoming and outgoing NRXN, NGL, CDH, NEGR, and contactin (CNTN) signaling (Fig. 6C, D). The ML microglia also had increased incoming EPHA signaling compared to MH and FL (Fig. 6C, D). These changes in NRXN, NGL, CDH, CNTN, NEGR, and EPHA signaling were ML-specific meaning signaling from these pathways was undetectable in the MH and FL microglia (Fig. 6C, D). Like the ML, FH microglia exhibited specific increases in NRXN, NEGR, and NGL incoming and outgoing signaling compared to MH and FL microglia, while incoming and outgoing NCAM (neuronal cell adhesion molecule) signaling was also increased (Fig. 6E, F).

We next examined the specific ligand-receptor pairs in the CDH, NRXN, NCAM, NEGR, SPP1, and EPHA pathways utilized by different cell types to communicate with microglia in the four samples. As seen in mature oligodendrocytes, multiple NRXN ligand-receptor pairs (NRNX3-NLGN1, NRXN1-NLGN1) were the dominant signaling molecules utilized by various cell types to signal to the ML and FH microglia (Fig. 6G). The NEGR1-NEGR1 ligand-receptor pair was also a prominent signaling pathway utilized by multiple cell types to communicate with FH microglia. NEGR1 is a cell adhesion molecule that plays a role in neuronal connectivity, learning, and social approach [89, 90]. CDH2-CDH2 ligand receptor pairs were also used by multiple cell types to signal to ML and FH microglia (Fig. 6G). CDH2 is a cell adhesion molecule that plays a role in axon guidance, synaptic function, neuronal cell death, and ASD [91]. Multiple EPHA ligand-receptor pairs were utilized by different cell types to signal exclusively to ML and FH microglia (Fig. 6G). CNTN ligand-receptor pairs (CNTN2-L1CAM, CNTN1-NRCAM) were also a source of signaling to ML and FH microglia (Fig. 6G). NCAMs are cell surface proteins that function in neurodevelopment and studies suggest play a role in ASD-related neuroinflammation [92]. Our results revealed different cell types utilized NCAM1-NCAM2 and NCAM1-NCAM1 to signal to ML and FH microglia (Fig. 6G). Finally, osteopontin (Spp1) is a cytokine involved in several physiological and pathophysiological processes including inflammation and macrophage activation [93, 94]. In FH, pericytes utilized Spp1-(ITGAV + ITGB5), Spp1-(ITGAV-ITGB1), and Spp1-(ITGA9 + ITGB1) ligand-receptor pairs to signal to microglia (Fig. 6G).

### Mature oligodendrocytes and microglial cells have distinct regulon activities that are driven by differences in sex and sociability in C58/J amygdala

The maintenance of cell identity involves the coordinated action of transcription factors (TFs) which regulate gene regulatory networks (GRNs) and control gene expression in cells. We utilized SCENIC to computationally reconstruct GRNs in mature oligodendrocytes and microglia separately based on single nuclei RNA expression data from C58/J amygdala to identify the key TFs that regulate these two cell types. SCENIC is comprised of three steps that include co-expression analysis, target gene motif enrichment analysis, and assessment of regulon activity [60]. SCENIC lists regulons that represent a TF and significantly enriched target genes, as well as regulon activity scores in each cell. We were particularly interested in determining whether these two cell types have different gene regulatory circuitries across our four groups of C58/J amygdala.

Our analysis indicates that GRN differences in C58/J amygdala mature oligodendrocytes are driven by differences in mouse sociability and sex. Following construction of the GRNs, we examined regulon activity scores in the mature oligodendrocytes in our four samples to determine the most active transcription factors. In addition, regulon activity scores in the dataset were binarized into “on/off” to further demonstrate the differences in transcription factor activity across the four groups of C58/J amygdala. Mature oligodendrocytes are myelin-forming cells that develop from neuroepithelial precursor cells in the ventricular zone of the central nervous system via precursors known as oligodendrocyte progenitor cells (OPCs) [95]. Transcription factors like Sox10 and Sox8 regulate oligodendrocytes and ensure proper lineage progression from OPCs to terminally differentiated myelinating oligodendrocytes. Sox10 is present at the pre-OPC stage and remains present as a key component of the regulatory network that controls oligodendrocyte development and terminal differentiation into mature oligodendrocytes [95]. During oligodendrocyte development, Sox8 accompanies Sox10 in the oligodendrocyte regulatory network [96]. Sox8 is less critical to oligodendrocyte development and differentiation compared to Sox10 [96]. However, Sox8 and Sox10 play equally important roles in myelin maintenance in mature oligodendrocytes [97].

**Fig. 7:**
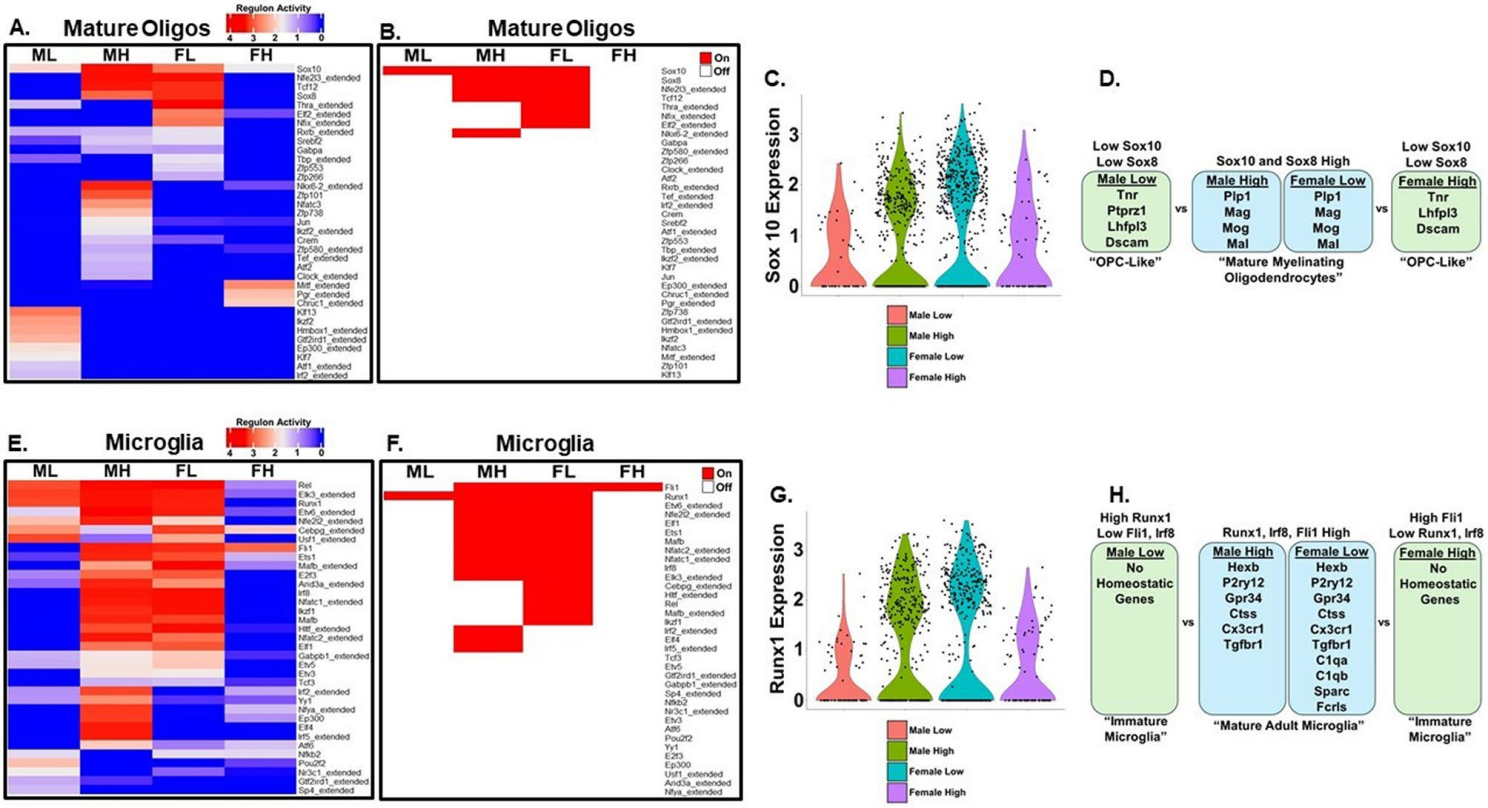
Mature oligodendrocytes and microglial cells have distinct regulon activities in C58/J amygdala that are dependent on mouse sex and sociability. Heatmaps of (**A, E**) average regulon activity scores and (B, F) binary regulon activity generated using SCENIC for mature oligodendrocyte (**A, B**) and microglia (**E, F**) clusters from amygdala of Male Low Social (ML), Male High Social (MH), Female Low Social (FL), and Female High Social (FH) C58/J mice (*n* = 1 mouse per group). For binary regulon activity, an AUC threshold of 0.7 was used such that red blocks in the heatmap represent regulons whose activity met that threshold and are “on”, while white blocks represent regulons with activity below this threshold and are “off”. (**C**) Violin plot of Sox10 expression in mature oligodendrocytes from representative C58/J samples. (**D**) Differentially expressed genes (DEGs) in ML, MH, FL, and FH mature oligodendrocytes that are regulated by Sox8 and Sox10. (**G**) Violin plot of Runx1 expression in microglia from representative C58/J amygdala samples. (**H**) DEGs in ML, MH, FL, and FH microglia that are regulated by Fli1, Irf8, and Runx1

Our network analysis revealed Sox10 was an active regulon in ML, MH, FL, and FH mature oligodendrocytes (Fig. 7A). However, Sox10 regulon activity was higher in MH (3.29 average regulon activity score) and FL (2.73 average regulon activity score) compared to ML (1.90 average regulon activity score) and FH (1.62 average regulon activity score) (Fig. 7A). The difference in Sox10 regulon activities between the four samples is especially evident when regulon activities were binarized which suggested Sox10 is “on” only in MH, FL, and ML mature oligodendrocytes (Fig. 7B). Sox10 expression was detected in ML, MH, FL, and FH mature oligodendrocytes (Fig. 7C). In ML and FH mature oligodendrocytes, Sox10-regulated genes were upregulated that are strongly associated with an “OPC identity” including *Tnr*, *Ptprz1*, *Lhfpl3*, and *Dscam* (Fig. 7D). In contrast, Sox10-regulated genes that were upregulated in MH and FL mature oligodendrocytes were associated with terminally differentiated myelinating oligodendrocytes such as *Plp1*, *Mag*, *Mog*, and *Mal* (Fig. 7D). Overall, the findings with Sox10 suggest mature oligodendrocytes in ML and FH differ from MH and FL in terms of lineage progression, differentiation, and maintenance of the differentiated state. Like Sox10, Sox8 was an active regulon in MH (2.89 regulon activity score) and FL (3.28 regulon activity score) mature oligodendrocytes (Fig. 7A, B). In contrast, Sox8 regulon activity was much lower in ML (0.32 regulon activity score) and FH (0.01 regulon activity score) mature oligodendrocytes (Fig. 7A, B). The important role that Sox8 plays in myelin maintenance in mature oligodendrocytes may explain why ML and FH mature oligodendrocytes look “OPC like” and less like mature myelinating oligodendrocytes compared to MH and FL [97].

GRN differences in C58/J amygdala microglia were also driven by differences in mouse sociability and sex. Microglial transcriptomes are tightly regulated by several key TFs that include Runx1, Irf8, and Fli1 [98, 99]. These three TFs play important roles in microglial differentiation into mature adult microglia which are characterized by increased expression of microglial homeostatic marker genes such as *Gpr34*, *P2ry12*, *Hexb*, *Fcrls*, *Ctss*, *C1qa*, *C1qb*, *Sparc*, and *Cx3cr1* [100]. Regulon activity for all three TFs was higher in MH (average regulon activity score: Runx1 3.30, Fli1 3.27, Irf8 3.29) and FL (average regulon activity score: Runx1 3.27, Fli1 3.23, Irf8 3.31) compared to ML (average regulon activity score: Runx1 3.08, Fli1 0, Irf8 0) and FH (average regulon activity score: Runx1 0, Fli1 2.88, Irf8 0) (Fig. 7E). The difference in regulon activities of the three TFs between the four samples is especially evident when regulon activities were binarized which suggested all three TFs were “on” only in MH and FL microglia, while only Fli1 was “on” in FH and only Runx1 was “on” in ML (Fig. 7F). Runx1 expression was detected in ML, MH, FL, and FH microglia (Fig. 7G). In ML and FH microglia, genes regulated by Runx1, Fli1, and Irf8 were upregulated that are microglia homeostatic genes and markers of mature adult microglia including *Hexb*, *P2ry12*, *Gpr34*, and *Ctss* (Fig. 7H). *Tgfbr1* was also upregulated in MH and FL microglia and it is known that Tgfb1 signaling is essential for microglia development and maturation [101]. In contrast, microglial homeostatic genes were not expressed in ML and FH microglia which suggests ML and FH microglia are at a stage of development that precedes microglial maturation (Fig. 7H).

### RNA velocity analysis reveals impaired differentiation of microglia and oligodendrocytes in ML and FH amygdala

**Fig. 8:**
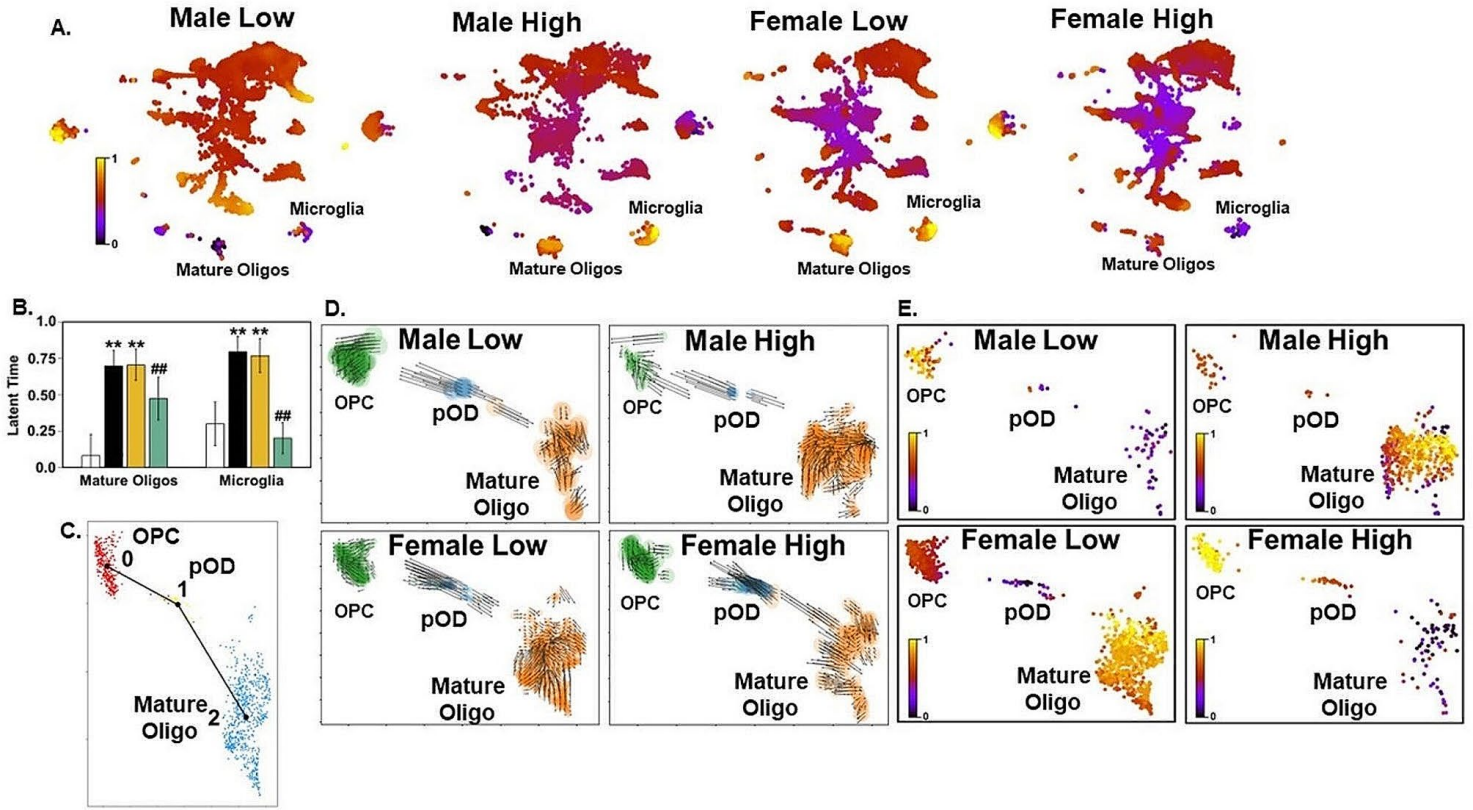
RNA velocity analysis reveals different developmental trajectories in OPCs and oligodendrocytes from C58/J amygdala that are dependent on mouse sex and sociability. (**A**) RNA velocity analysis latent time for Male Low Social (ML), Male High Social (MH), Female Low Social (FL), and Female High Social (FH) amygdala cell types projected on to a UMAP plot. (**B**) Graph of latent time in mature oligodendrocytes and microglia from ML, MH, FL, and FH amygdala. (**C**) Diagram of predicted developmental dynamics of OPCs, pre-oligodendrocytes (pOD), and mature oligodendrocytes where a cell differentiation trend moves from OPCs (0 or initial cluster) to pre-oligodendrocytes [1] to mature oligodendrocytes [2]. (**D**) RNA velocities of OPCs, pre-oligodendrocytes, and mature oligodendrocytes from ML, MH, FL, and FH amygdala with arrows showing direction of development. (**E**) RNA velocity analysis latent time for OPCs, pre-oligodendrocytes, and mature oligodendrocytes from ML, MH, FL, and FH amygdala. Results reported as mean ± SEM (*n* = 1 mouse per group), **p < 0.01 vs. ML, ##*p* < 0.01 vs. FL & MH using Student’s t-test

We performed RNA velocity analysis to investigate the transition states of oligodendrocytes and microglia in C58/J amygdala [53, 54]. Latent time is a measure that is based on a cell’s transcriptional dynamics and is used to predict the time required for a cell to differentiate with longer latent times indicating cells are more differentiated. FL and MH mature oligodendrocytes and microglia demonstrated greater latent times compared to FH and ML (Fig. 8A-B). Given our findings in mature oligodendrocytes, we examined the transition states of OPCs, pre-oligodendrocytes (pODs), and mature oligodendrocytes to see if these transitions differed between our samples. Studies have demonstrated that oligodendrocyte cells are ordered along a differentiation trajectory with OPCs as the initial state followed by pODs and then mature oligodendrocytes (Fig. 8C) [102]. As shown in Fig. 8D, the RNA velocity vector field for our four samples overlaid on the UMAP indicates that MH and FL mature oligodendrocyte velocity vectors are shorter and pointing away from OPCs and pODs indicating the cells are in homeostasis. In contrast, the vectors in the FH and ML mature oligodendrocytes are longer indicating they are still undergoing rapid differentiation and many of the vectors are pointing away from the mature oligodendrocyte cluster and in many instances towards the pOD cluster which suggests many of the cells are still pre-myelinating oligodendrocytes (Fig. 8D). Furthermore, all the vectors in the FH pODs are long and are pointing towards the OPCs and away from the mature oligodendrocytes indicating most cells in that cluster are still OPC-like (Fig. 8D). Interestingly, the velocity vectors in the OPC cluster of all four samples are short in length suggesting OPCs are quiescent and that the lack of mature oligodendrocytes in the ML and FH is not due to a lack of OPCs (Fig. 8D). Overall, RNA velocity vector field analysis suggests a sequential commitment from OPCs to pODs and then finally mature oligodendrocytes in FL and MH, while there appears to be very few mature oligodendrocytes in ML and FH which may be due to disruption of pOD differentiation. In Fig. 8E, latent time analysis of the three clusters further suggests that there are very few terminally differentiated mature oligodendrocytes in ML and FH compared to FL and MH, while many OPCs in each sample are quiescent.

RNA velocity analysis also enabled us to investigate the transcriptional dynamics of driver genes during cellular state transition. Supplemental Fig. 10 shows heatmaps of specific lineage driver genes whose relative expression levels are ordered along latent time. In Supplemental Fig. 10C, the gene trend plots in FL follow the predicted expression trajectory of cluster-specific marker genes for OPCs (*Gria4*, *Plpp4*) that eventually differentiate into oligodendrocytes (*Rffl*). In contrast, the predicted expression trajectory of cluster-specific markers is reversed in ML and FH (Supplemental Fig. 10A, D). For instance, in FH the latent time shows OPCs have a longer latent time and are quiescent compared to mature oligodendrocytes which have a shorter latent time (Supplemental Fig. 10D). As a result of this, the early driver genes like *Bcas1os2* (marker of early myelinating oligodendrocytes), *Enpp6* (early marker of oligodendrocyte differentiation), and *Pld1* (marker of the morphological differentiation of oligodendrocytes prior to myelination) are associated with mature oligodendrocytes and differentiating oligodendrocytes rather than OPCs (Supplemental Fig. 10D).

## Discussion

The focus of the present study was on the long-term, persistent transcriptomic and epigenetic changes that could underlie divergent social phenotypes and effects of subchronic oxytocin treatment. Our comparison of the C57BL/6J and C58/J inbred strains identified transcriptional and pathway signatures that suggest immune-related biological processes differ in amygdala cells from C58/J, a model of ASD-like behavior. We identified differentially hyper- and hypomethylated regions in genes in C58/J amygdala when compared to C57BL/6J amygdala. snRNA-Seq data from the C58/J amygdala identified a consistent transcriptional signature in mature oligodendrocytes and microglia characterized by altered ASD risk gene expression and impaired expression of myelin-related genes and microglial homeostatic genes that was dependent on sex and sociability. Alterations in gene regulatory networks, impaired cell-to-cell communication, and aberrant cell differentiation were identified in microglia and mature oligodendrocytes that may help explain why a reduced density of both cell types was observed in ML and FH C58/J amygdala. Many of these features were verified in our Bulk RNA-Seq data from C58/J amygdala. Taken together, these results provide preclinical evidence that defects in mature oligodendrocytes and microglia play an important role in sociability which is one of the core deficits of ASD.

### The C58/J mouse model is a useful preclinical tool for understanding the mechanistic basis of sociability deficits

The C58/J strain has several behavioral features that reflect symptoms of ASD, including a lack of social preference in the 3-chamber social choice test, hyperactivity, and abnormal repetitive responses [37, 103–105]. A previous study has shown that in contrast to C57BL/6J mice, C58/J and other low sociability strains display divergent phenotypes, such that approximately 50% of mice have positive sociability while 50% show marked social avoidance [36]. This provides an opportunity to investigate both genetic risk for ASD-like social deficits, through a between-strain comparison with C57BL/6J, and risk based on differences in sociability, by within-strain comparisons between high sociability and low sociability isogenetic C58J mice.

Our studies focused on the amygdala which is a brain region that plays a key role in ASD. In animals, the amygdala is composed of multiple subnuclei which are organized into three groups that include the basolateral amygdala (BLA) group, the centromedial amygdala (CM) group, and the cortical amygdala group with each having different functions and connections [106]. Human amygdala also consists of multiple subnuclei which can be divided into subregions such as BLA and CM [107, 108]. Studies in humans have demonstrated regions such as the BLA and CM play an important role in emotional and social experiences, repetitive behaviors, as well as pain sensitivity, while subnuclei research is actively being done to better understand amygdala function [108]. Interestingly, studies have revealed adolescents with ASD have enlarged BLA and CM volumes compared to typically developing adolescents, while an association was identified between increased amygdala subnuclei volume and ASD symptomology [107]. In our investigation, the entire amygdala was dissected and used for Bulk RNA-Seq and snRNA-Seq. Thus, we did not compare amygdala subnuclei. Given the findings that amygdala subnuclei possess unique functions and that enlarged subnuclei are associated with ASD core features it is plausible differences in gene expression may exist between subnuclei in C58/J mice that depend on sex, sociability, and strain.

Bulk RNA-Seq analysis of amygdala from C58/J and C57BL/6J mice revealed immune-related genes and pathways were downregulated in C58/J amygdala compared to C57BL/6J. Alterations in the immune system are a major factor contributing to the pathogenesis of ASD [68]. Pathways were downregulated in C58/J compared to C57BL/6J that are related to regulation of IL-1β and TNFα production, regulation of adaptive immunity, and regulation of T-cell immunity which other studies have shown to be dysregulated in ASD [68, 109]. Several immune-related genes were differentially expressed between the strains that play a role in ASD including *Pla2* genes, innate immunity genes, and the axon guidance molecule *Robo4*. In addition to Bulk RNA-Seq analysis, our differential methylation analysis suggests C58/J and C57BL/6J mice have distinct epigenetic profiles. Overall, the between-strain comparison indicates that C58/J has transcriptional profiles with significant alterations in multiple immune-related genes and studies suggest immune system problems are involved in the pathogenesis of ASD.

Our snRNA-Seq analysis of the C58/J amygdala provided confirmation of our Bulk RNA-Seq data and our findings in the ML group were particularly interesting given the higher prevalence of ASD diagnoses in males. The ML differed from both the MH (based on sociability) and the FL (based on sex) via upregulation of SFARI ASD risk genes as well as ASD-related pathways in microglia and mature oligodendrocytes. Similar to across-strain changes identified by our comparison of C58/J and C57BL/6J, ML microglia exhibited down regulation of several immune-related pathways including Toll-Like receptor 2 signaling, cytokine production, MAPK signaling, myeloid cell activation, and microglial activation in comparison to the MH and FL groups. Our data also indicate microglia cell numbers were reduced in ML and FH compared to MH and FL. RNA velocity and SCENIC analyses revealed aberrant microglia differentiation in ML and FH, while cell-to-cell interaction analysis indicated upregulated Neurexin and Ephrin signaling which are known to stimulate cell differentiation and regulate synapse formation [78, 79, 110].

These results are significant due to the important role that microglia play in the central nervous system and because microglial abnormalities may cause many of the pathological phenotypes of ASD. Microglia are immune cells that maintain homeostasis in the central nervous system via their role in immune defense, neurogenesis, synapse formation, and synaptic pruning [111, 112]. Thus, maintaining the normal number and function of microglia is critical to the health of the central nervous system. Mouse models have shown damage to microglia affect synaptic pruning that leads to deficits in social behavior, while synapse formation in mice was also affected after microglia loss [112]. In the Ptenm3m4/m3m4 mouse model, the ASD risk gene phosphatase and TENsin homolog (PTEN) is only expressed in the cytoplasm which results in ASD-like features in mice [112, 113]. Furthermore, Ptenm3m4/m3m4 mice had elevated levels of activated microglia which increased microglial phagocytic activity and led to abnormal synaptic pruning which affected neural development [113]. In contrast, it has been shown that Transmembrane protein 59 (TMEM59) expression is decreased in ASD patients, while knocking out TMEM59 in mouse microglia resulted in mice with ASD-like behaviors, defective microglia, defective synaptic pruning, and enhanced excitatory neurotransmission [114]. Downregulation of CD93 in microglia of mice also impaired synapse engulfment and synaptic pruning [114]. In the Cntnap2 ASD mouse model, microglial activation was increased which inhibited the development of neural circuits involved in sociability due to excessive synaptic pruning [115]. Interestingly, oxytocin administration rescued many of the social deficits in the Cntnap2 ASD mouse model [116]. Given what is known about the role of microglia in ASD, it is possible that the reduced numbers of ML and FH microglia could contribute to altered immune signaling and would have negative implications for synaptic pruning, synapse formation, and cell clearance. Further, the reduced number of microglia may have effects on oligodendrogenesis and myelination. Research has shown that microglia can promote the survival and differentiation of oligodendrocytes, support myelination in the nervous system, and ensure myelin development [117, 118].

### Myelination defects in the C58/J mouse model

Evidence exists that supports a role for oligodendroglial cells and myelination in ASD. Oligodendrocytes may play a critical role in the pathophysiology of ASD, because they myelinate axons that allow for proper axonal nerve conduction in the whiter matter of the brain. Our snRNA-Seq studies revealed mature oligodendrocytes from ML amygdala exhibit downregulation of myelin-related genes and pathways compared to MH (sociability) and FL (sex). ML mature oligodendrocytes also exhibited reduced cell number compared to FL and MH mature oligodendrocytes. RNA velocity analysis and cell-to-cell interaction analysis revealed differentiation is impaired in ML mature oligodendrocytes and several pathways that influence oligodendrocyte differentiation were altered in ML including Gpr37-PSAP, SEMA6A, SEMA6B, Neurexin, and Ephrin signaling. GRN analysis revealed an absence of *Sox8* and *Sox10* regulon activity in ML. *Sox10* and *Sox8* are critical to myelination and appear to drive expression of DEGs that promote myelin formation in MH and FL mature oligodendrocytes [97].

Studies in humans and in mouse models support our findings in the C58/J mouse model regarding oligodendrocytes and myelination. Individuals with ASD show atypical growth in various white matter brain regions throughout life [119]. Axon tracts in regions like the external capsule show hypomyelination during the first fifteen years of life, while the hippocampus exhibits hypermyelination [119]. Myelin alterations in these brain regions tend to normalize later in life, while brain regions such as the cerebellum and internal capsule remain hypomyelinated [119]. Multiple mouse models of ASD have reported impaired myelination. Tuberous sclerosis complex 1 (*Tsc1*) knockout mice display hypomyelination and reduced oligodendrocyte density [120], while fragile X (Fmr1) knockout mice display delayed myelination and reduced OPC density in cerebellum [121]. Mouse mutants for the ASD risk gene *Cntnap2* also exhibit delayed myelination [122].

*Tcf4* mutant mice are an ASD mouse model with oligodendrocytes that display a similar profile compared to C58/J ML and FH mature oligodendrocytes [123]. Specifically, mutation in the *Tcf4* gene resulted in reductions in genes involved in myelin formation, reductions in mature oligodendrocyte numbers, but no change in OPC density [123]. Single cell analysis of human control and ASD cells from cortical brain tissue have previously revealed reduced mature oligodendrocytes and microglial cell density in ASD samples while no change in OPC cell density was found [123, 124]. Interestingly, TEM imaging showed *Tcf4* mutant mice display reduced mature oligodendrocytes in the corpus callosum as well as a reduction in myelinated axons [123]. Several other neuroimaging studies have also reported white matter deficiency in ASD patients [125]. However, our MRH analysis revealed no differences in connectomes or brain volumes between our male and female low and high social C58/J brains. It is important to note that hypomyelination and reduced mature oligodendrocyte density is not a universal finding in mouse ASD models. *Pten* knockout mice display hypermyelination in brain, while knockdown of *MeCP2* in primary rat oligodendrocytes increased synthesis of genes associated with myelin synthesis (*Plp1*, *Mog*, *Mbp*) [126, 127]. Studies have also shown that ASD-related myelination deficits are region-specific and that some brains from the same human ASD patient have shown opposite changes in myelination in different brain regions such as prefrontal cortex and cerebellum [128]. What is clear, however, is that altered oligodendrocyte biology and myelination play an important role in ASD.

### An ASD-like, myelin-deficient, inflammatory transcriptomic profile in FH C58/J

A surprising finding in our studies was that an ASD-like transcriptome profile was discovered in FH mature oligodendrocytes and microglia. Given that ASD prevalence is elevated in males and is defined by poor social skills and functioning, one would predict MH (sex) and FL (sociability) mature oligodendrocytes and microglia would look more ASD-like when compared to those same cell types in FH. Our data revealed that this was not the case for our analysis of C58/J. In support, our Bulk RNA-Seq analysis revealed significant differences in ASD-related biological pathways between MH and FH amygdala. Moreover, the Bulk RNA-Seq data confirmed that MH amygdala exhibited upregulation of pathways related to myelin assembly and axon ensheathment when compared to FH amygdala. This finding validated our discovery that FH mature oligodendrocytes exhibit downregulated pathways associated with myelination and axon ensheathment compared to MH mature oligodendrocytes.

Interestingly, our Bulk RNA-Seq data also showed oxytocin administration upregulated biological pathways related to myelin assembly, axon ensheathment, and oligodendrocyte differentiation in FH amygdala. This action could have been the basis for the prosocial oxytocin effects previously reported in C58/J and other mouse models [39, 40]. These data also provide potential insight into the mechanisms by which oxytocin, as a potential ASD therapeutic, might function with respect to rectifying deficiencies in myelin-related pathways and signaling in mature oligodendrocytes within the FH amygdala. Leading edge analysis of Bulk RNA-Seq data in FH oxytocin-treated amygdala also identified several genes (e.g., *Opalin*) whose expression was increased in FH oxytocin-treated amygdala that play a role in oligodendrocyte differentiation (Supplemental Fig. 11A). This finding supports our cell-to-cell interaction data and RNA velocity data that suggest oligodendrocyte differentiation is impaired in FH mature oligodendrocytes which contributes to reduced FH mature oligodendrocyte cell density.

While our research group and others have reported that repeated oxytocin treatment can lead to increased sociability in mouse models [16, 38, 39, 129], not all investigators have observed prosocial effects with chronic oxytocin regimens [130]. Results from clinical studies examining chronic oxytocin effects in ASD have been similarly mixed [131–133]. In the present study, the subchronic treatment with oxytocin did not lead to increased sociability and did not alter patterns of divergent sociability in either male or female mice (Supplemental Fig. 1). As mentioned above, oxytocin treatment was associated with an intriguing upregulation of genes linked to myelin synthesis in high sociability female mice. Although the negative results from the behavioral test limit our ability to link the transcriptional changes to functional consequences, our findings suggest that exogenous oxytocin might have effects on myelin remodeling within specific brain regions.

In addition to aberrant myelination, the FH is also interesting because of its inflammatory profile. Bulk RNA-Seq data showed genes (*Tlr1*, *Il15ra*, *Edn1*) and pathways were upregulated in FH amygdala compared to MH amygdala that are associated with the immune system (Supplemental Fig. 9B-D, Supplemental Fig. 11B), while oxytocin treatment also decreased pathways associated with detoxification of reactive oxygen species and programmed cell death in FH amygdala (Supplemental Fig. 11C). These findings in the Bulk RNA-Seq data support snRNA-Seq data in FH that pericytes signal to microglia via the proinflammatory Spp1 pathway, while other cell types signal to microglia via the proinflammatory NCAM pathway. Interestingly, a study in adult male C58/J mice revealed increases in microglial density and pro-inflammatory interferon gamma and monocyte chemoattractant protein 1 in hippocampus, along with reduced levels of the anti-inflammatory enzyme arginase 1 compared to C57BL/6J mice which suggests elevated inflammation [134]. Our findings in FH raise questions about sex-specific differences in clinical presentation and diagnosis between males and females with ASD. The fact that ML and FH samples are the most ASD-like suggests ASD symptoms and presentations may be different in males and females regarding at least one aspect of typical ASD core deficits like sociability and this may be why females are underdiagnosed [135]. One possibility is that amygdala plays an inhibitory role to limit risky or impulsive social approach to unfamiliar strangers, and alterations in amygdala function could lead to abnormal hypersociability in normally cautious female mice.

### Limitations

Our findings are significant. However, several caveats can be noted. We have only investigated a single model of ASD-like behavior, and a single aspect (social approach) of the complex behavior domain. Further work is needed to determine whether our findings generalize to a broader range of mouse models of genetic and environmental ASD risk, whether similar epigenetic signatures in brain underlie other social phenotypes, and the contribution of brain regions, other than amygdala, to ASD-like social deficits. The cellular mechanisms linking reduced oligodendrocyte differentiation and reduced myelination to an ASD phenotype in C58/J mice needs further investigation. Additional snRNA-Seq studies would be needed to determine if effects in oligodendrocytes and microglia are unique to amygdala or if this occurs in other brain regions. In our study, the subchronic oxytocin regimen did not shift the pattern of social divergence in the C58/J model (Supplemental Fig. 1A-D). Thus, the effects of oxytocin need further examination to better understand its potential as an ASD therapeutic. Cell proportion estimates may differ between ML, MH, FL, and FH amygdala in snRNA-Seq experiments due to slight differences in where the tissue originated when dissected. We did not assess the estrous cycle in our studies which is an important biological variable that can help understand the mechanisms responsible for behavioral and molecular differences between male and female mice [136]. For the Bulk RNA-Seq studies, we acknowledge there is a small window of time between induction of anesthesia and effective cooling of brain tissue during perfusion where tissue can undergo hypoxia and oxidative stress which can activate endogenous RNases that can cause changes in differential gene expression in the rodent transcriptome [137]. Finally, the small sample size (*n* = 1 per group) in the snRNA-Seq studies reduces the statistical power of the study, hinders the reliability of our results, and makes interpretation and generalizability of the results difficult.

## Conclusions

In summary, our work demonstrates the utility of the C58/J mouse model in evaluating the influence of sex and sociability on the transcriptome in brain regions that play an important role in ASD. Our single-nucleus transcriptome analysis elucidates the pathological roles of oligodendrocytes and microglia from amygdala in ASD. Our study provides many details regarding specific regulatory features that are disrupted in these two cell types that include transcriptional gene dysregulation (ASD risk genes, myelinating genes, microglial homeostatic genes), aberrant cell differentiation, impaired gene regulatory networks, and alterations in key pathways that promote microglia and oligodendrocyte differentiation. Our work also reveals the cellular changes oxytocin administration produces within cells of the brain. Future research is needed to determine if targeting oligodendrocytes and microglia could be developed into effective treatments for ASD. Research at the single cell level should certainly provide more potential ASD therapeutic targets.

### Electronic supplementary material

Below is the link to the electronic supplementary material.

Supplementary Material 1

Supplementary Material 2

## Data Availability

The corresponding author will make the datasets used and analyzed in this paper available upon request. The datasets will also be made publicly available following acceptance for publication.

## Abbreviations

ASD: Autism Spectrum Disorder
Bulk RNA-Seq: Bulk RNA-Sequencing
DEG: Differentially Expressed Gene
FH: Female High Social
FL: Female Low Social
GRN: Gene Regulatory Network
MH: Male High Social
ML: Male Low Social
snRNA-Seq: single nucleus RNA-Sequencing
