# Supplementary material for: Transcriptome analysis identifies an ASD-Like phenotype in oligodendrocytes and microglia from C58/J amygdala that is dependent on sex and sociability
Source: Behav Brain Funct. 2024 Jun 19;20:14. doi: 10.1186/s12993-024-00240-3 (PMC11188533; doi:10.1186/s12993-024-00240-3)

**Supplemental Figure 1**. Lack of oxytocin effects on divergent phenotypes in C58/J mice. (A-D) Comparison of social preference based on time spent in close proximity to a stranger mouse during the 3-chamber social choice test in male and female C58/J high social and low social mice that received vehicle or oxytocin (OXY). Data are mean ± SEM time spent within 5.0 cm proximity to a cage containing a stranger mouse, or an empty cage. N=8-10 mice per group. *p<0.05, **p<0.01, ***p<0.001, ns (nonsignificant); within-group repeated measures comparison, of side.

**Supplemental Figure 2**. Images produced from Magnetic Resonance Histology (MRH) of C58/J mouse brain. The MRH processing pipeline produced a series of images in which the signal highlights different diffusion parameters and brain anatomy. The figure shows representative images from the average of a C58/J Male High Social mouse where Image A shows Mean Diffusivity (MD), Image B shows fractional anisotropy (FA), and Image C shows color fractional anisotropy (cIrFA). The scale bar is 1 mm.

**Supplemental Figure 3**. Glutamatergic cell markers in C58/J mouse amygdala. Dotplots of (A,B) Glutamatergic cell clusters showing percentage of cells within each cluster derived from amygdala taken from C58/J male and female mice and marker gene expression in each cluster. Dot size indicates percentage of cells within a cluster, while color shows average expression level across all cells within a cluster.

**Supplemental Figure 4**. GABAergic cell markers in C58/J mouse amygdala. Dotplots of (A,B) GABAergic, and (E) Non-Neuronal cell clusters showing percentage of cells within each cluster derived from amygdala taken from C58/J male and female mice and marker gene expression in each cluster. Dot size indicates percentage of cells within a cluster, while color shows average expression level across all cells within a cluster.

**Supplemental Figure 5**. Non-Neuronal cell markers in C58/J mouse amygdala. Dotplots of Non-Neuronal cell clusters showing percentage of cells within each cluster derived from amygdala taken from C58/J male and female mice and marker gene expression in each cluster. Dot size indicates percentage of cells within a cluster, while color shows average expression level across all cells within a cluster.

**Supplemental Figure 6**. Percentage of 29 cell types across C58/J amygdala depends on sex and sociability. Percentage of (A) non-neuronal and GABAergic and (B) glutamatergic cell types in Male Low Social, Male High Social, Female Low Social, and Female High Social amygdala (n = 1 mouse per group).

**Supplemental Figure 7**. Effect of sociability on differential gene expression in C58/J amygdala. (A-B) Bar graphs showing total differentially expressed genes (DEGs) and SFARI ASD risk DEGs in whole amygdala and in individual cell types when C58/J mouse groups were compared based on sociability (n = 1 mouse per group). DEGs in whole amygdala were obtained via bulk RNA-Seq, while DEGs in individual cell types were obtained via snRNA-Seq. For Bulk RNA-Seq analysis, genes with an adjusted *P*-value < 0.05 and Log_2_ Fold Change > 0.5 and < -0.5 were considered differentially expressed. For snRNA-Seq analysis, genes with an adjusted *P*-value < 0.05 and Log_2_ Fold Change of > 0.5 and < -0.5 were differentially expressed.

**Supplemental Figure 8**. Effect of sex on differential gene expression in C58/J amygdala. (A-B) Bar graphs showing total differentially expressed genes (DEGs) and SFARI ASD risk DEGs in whole amygdala and in individual cell types when C58/J mouse groups were compared based on sex (n = 1 mouse per group). DEGs in whole amygdala were obtained via Bulk RNA-Seq, while DEGs in individual cell types were obtained via snRNA-Seq. For Bulk RNA-Seq analysis, genes with an adjusted *P*-value < 0.05 and Log_2_ Fold Change > 0.5 and < -0.5 were considered differentially expressed. For snRNA-Seq analysis, genes with an adjusted *P*-value < 0.05 and Log_2_ Fold Change of > 0.5 and < -0.5 were differentially expressed.

**Supplemental Figure 9**. GSEA analysis of Male High Social versus Female High Social amygdala comparison using Bulk RNA-Sequencing data. (A) Results of Gene Ontology (GO) enrichment analysis of pathways involved in ASD pathogenesis, (B) Results of GO enrichment analysis of immune-related pathways, (C) Results of REACTOME pathway analysis, and (D) Results of HALLMARK enrichment analysis. Positive normalized enrichment score (NES) indicates pathways are upregulated in group indicated above each graph. The significantly enriched pathways were defined by adjusted *P*-value < 0.05 and FDR < 0.25.

**Supplemental Figure 10**. Heatmap of gradual gene expression changes along the latent time trajectory in C58/J amygdala OPCs, differentiating oligodendrocytes, and mature oligodendrocytes. Each column shows gene expression levels in the indicated cell type at each point in latent time and each row represents a corresponding gene in (A) Male Low Social, (B) Male High Social, (C) Female Low Social, and (D) Female High Social amygdala (n = 1 mouse per group). The gradient from blue to yellow indicates the amount of gene expression from lowest to highest.

**Supplemental Figure 11**. GSEA identifies differentially expressed REACTOME pathways from Bulk RNA-Seq data generated in Female High Social vehicle-treated (FH) amygdala when compared to Female High Social oxytocin-treated amygdala (FHOXY). (A) Differentially expressed genes that were identified as leading edge genes following GSEA Gene Ontology Biological Process (GO BP) pathway analysis of Bulk RNA-Seq data from FH and FHOXY amygdala. Positive log2FC indicates genes were upregulated in FHOXY. Genes with an adjusted *P*-value < 0.05 and Log_2_ Fold Change (log2FC) > 0.5 and < -0.5 were considered differentially expressed. (B) Differentially expressed genes that were identified as leading edge genes following GSEA HALLMARK pathway analysis of Bulk RNA-Seq data from MH and FH amygdala. Positive log2FC indicates genes were upregulated in FH. Genes with an adjusted *P*-value < 0.05 and Log_2_ Fold Change (log2FC) > 0.5 and < -0.5 were considered differentially expressed. (C) REACTOME enrichment analysis of pathways in FH vehicle-treated amygdala compared to FHOXY amygdala obtained by Bulk RNA-Seq analysis and GSEA (n = 4-5 mice per group). Positive normalized enrichment score (NES) indicates pathways are upregulated in the FH group. The significantly enriched pathways were defined by adjusted *P*-value < 0.05 and FDR < 0.25.

Supplemental Figure 1


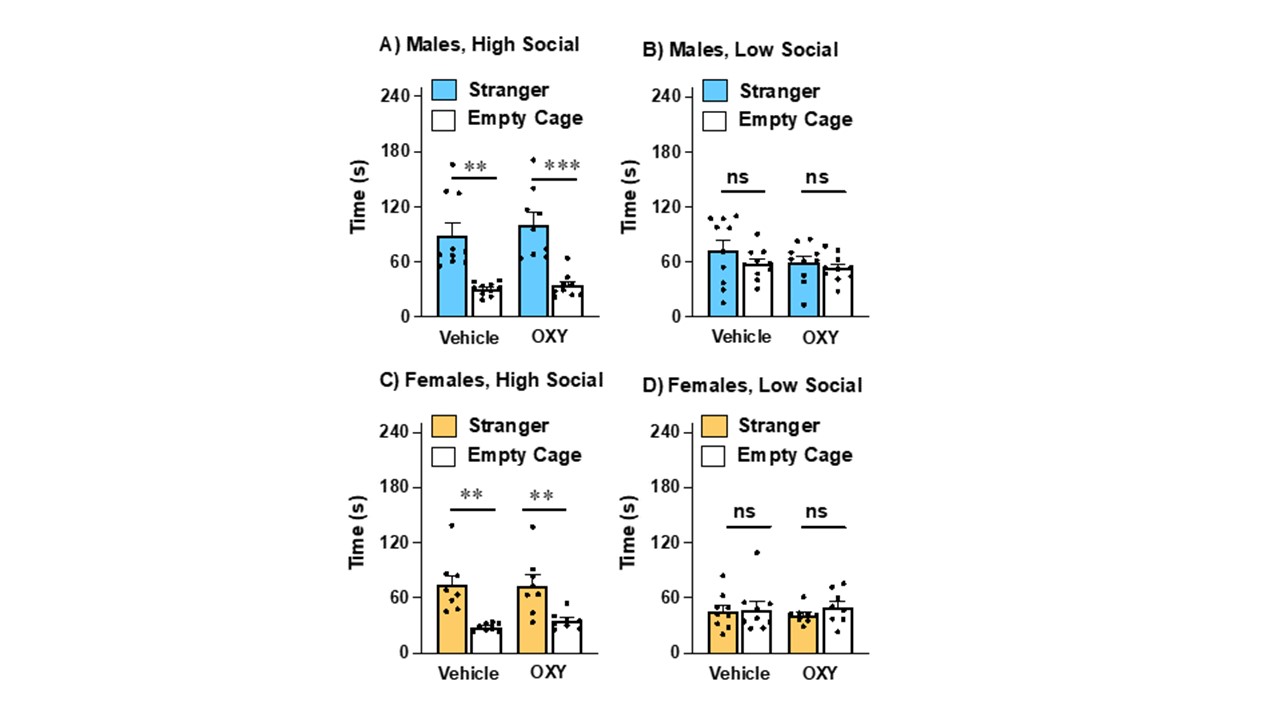


Supplemental Figure 2


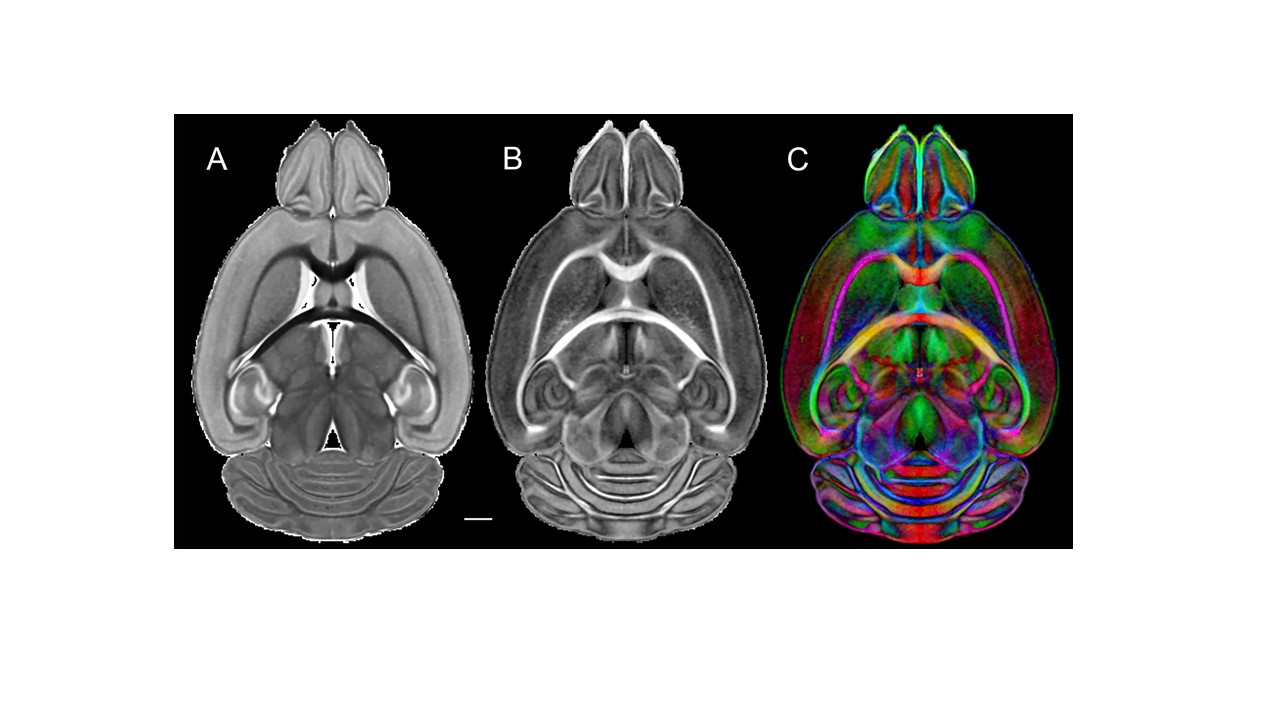


Supplemental Figure 3


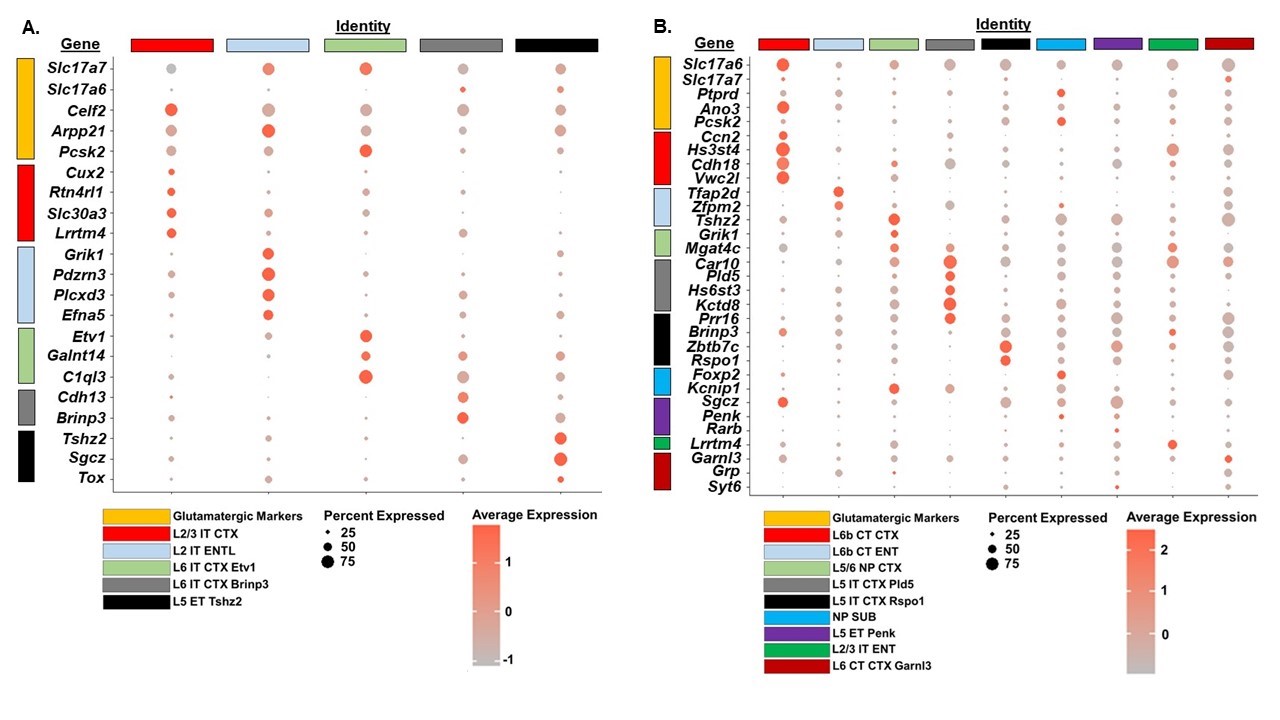


Supplemental Figure 4


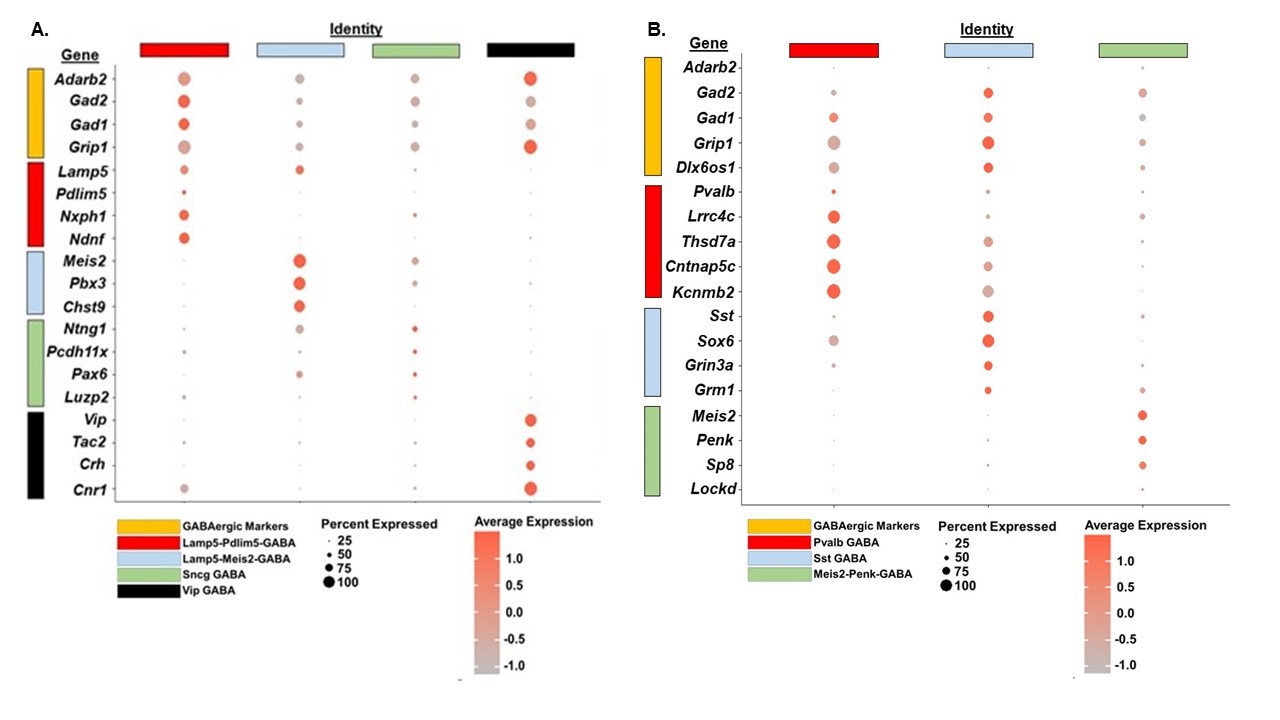


Supplemental Figure 5


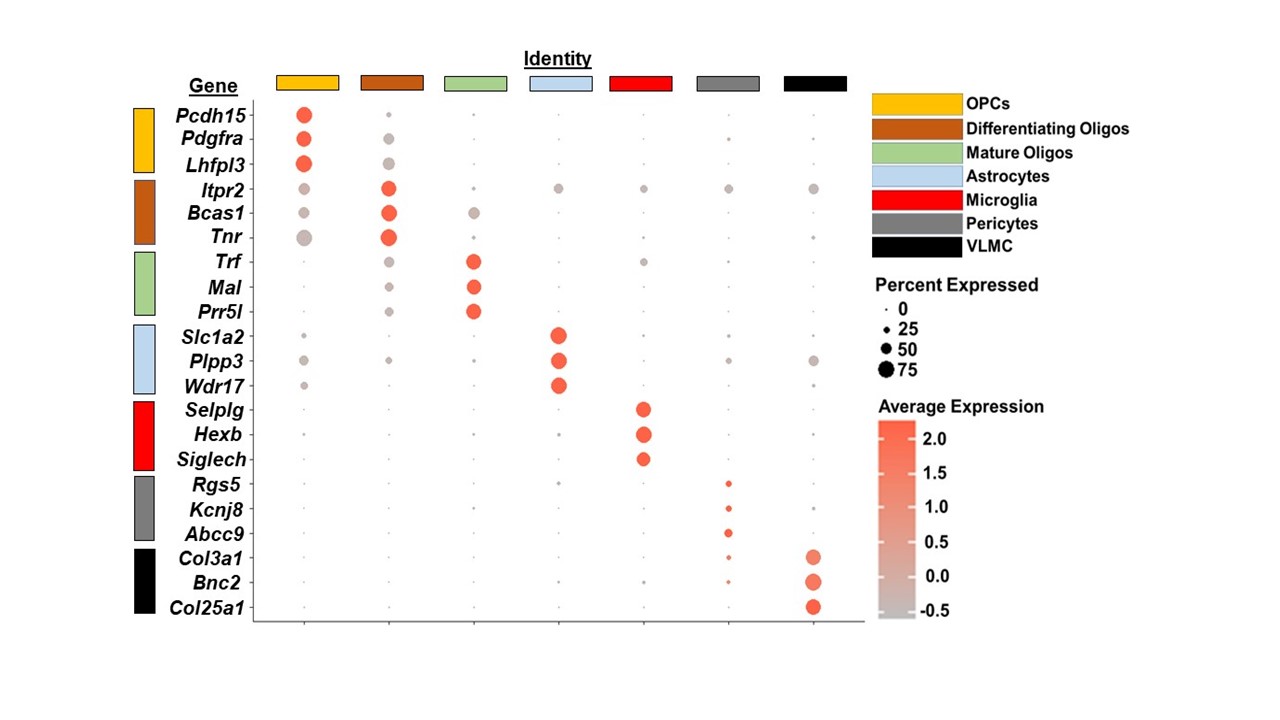


Supplemental Figure 6


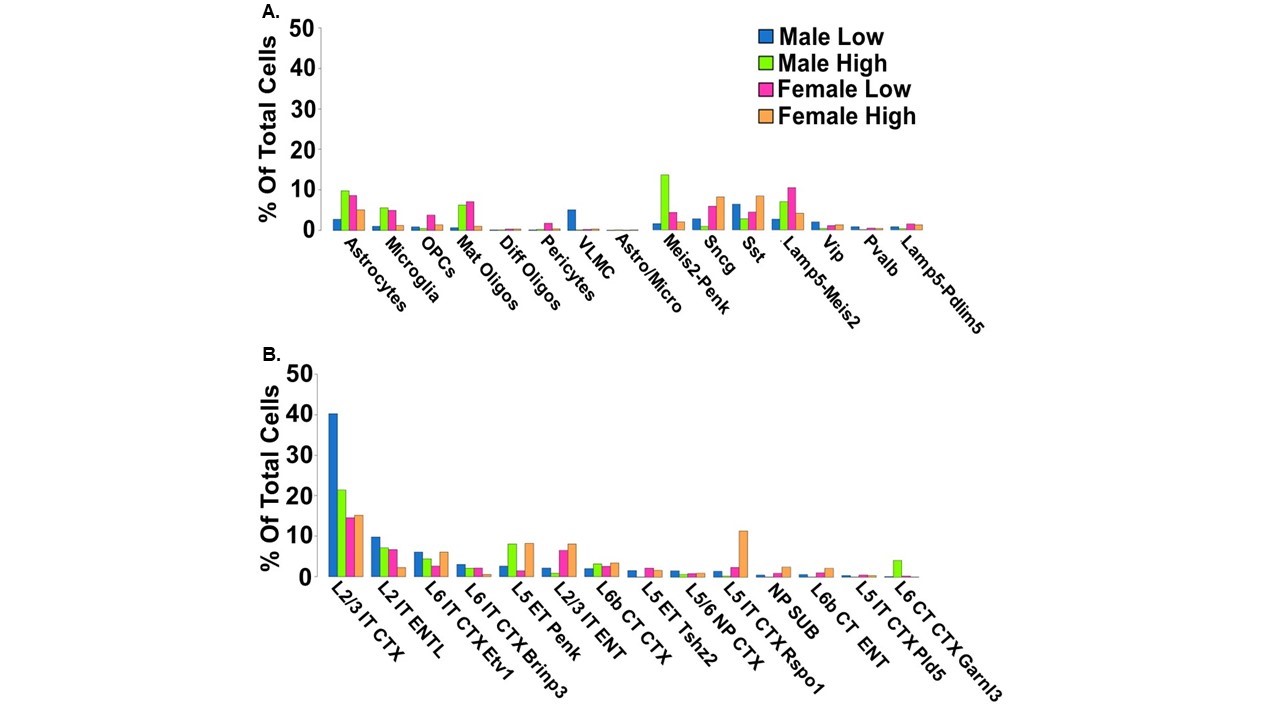


Supplemental Figure 7


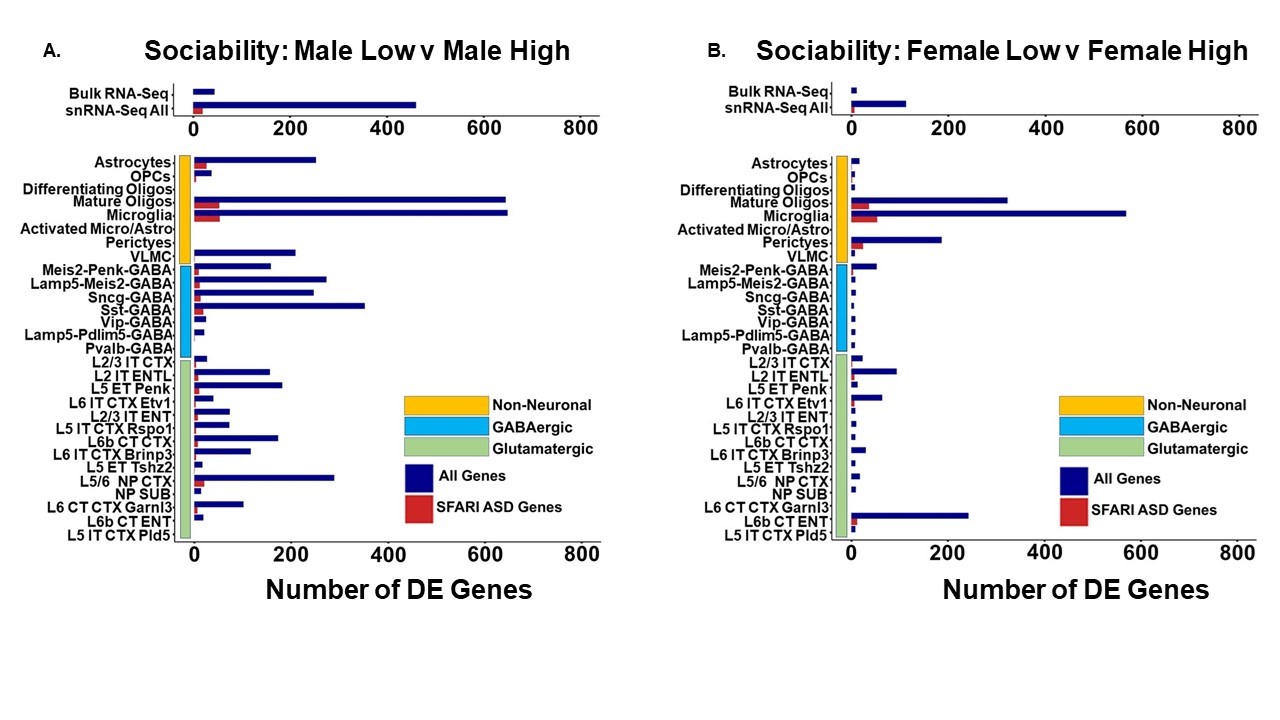


Supplemental Figure 8


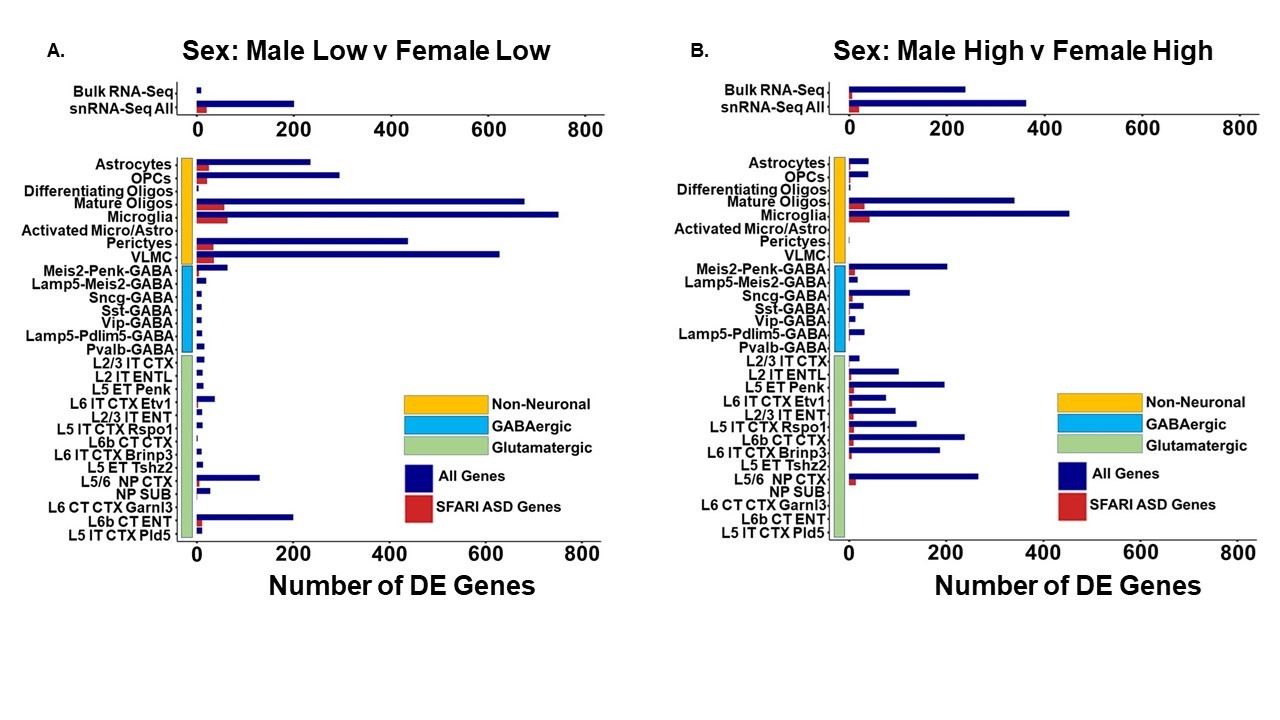


Supplemental Figure 9


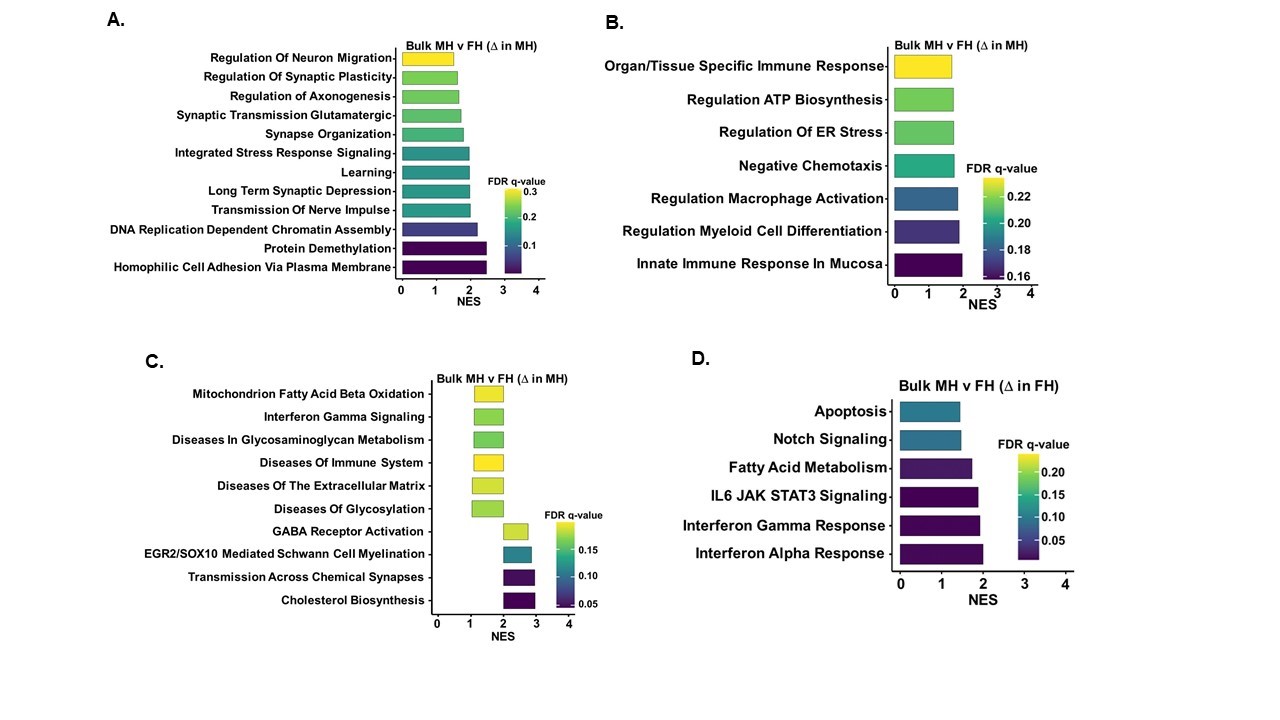


Supplemental Figure 10


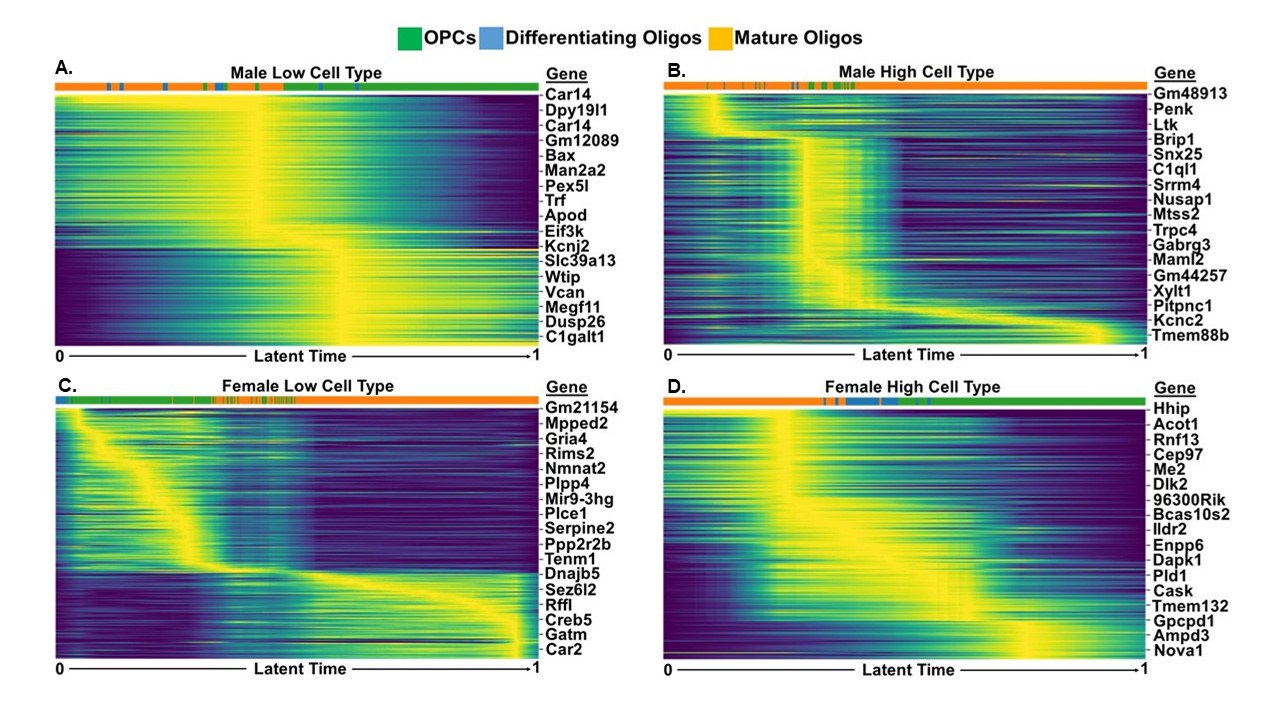


Supplemental Figure 11


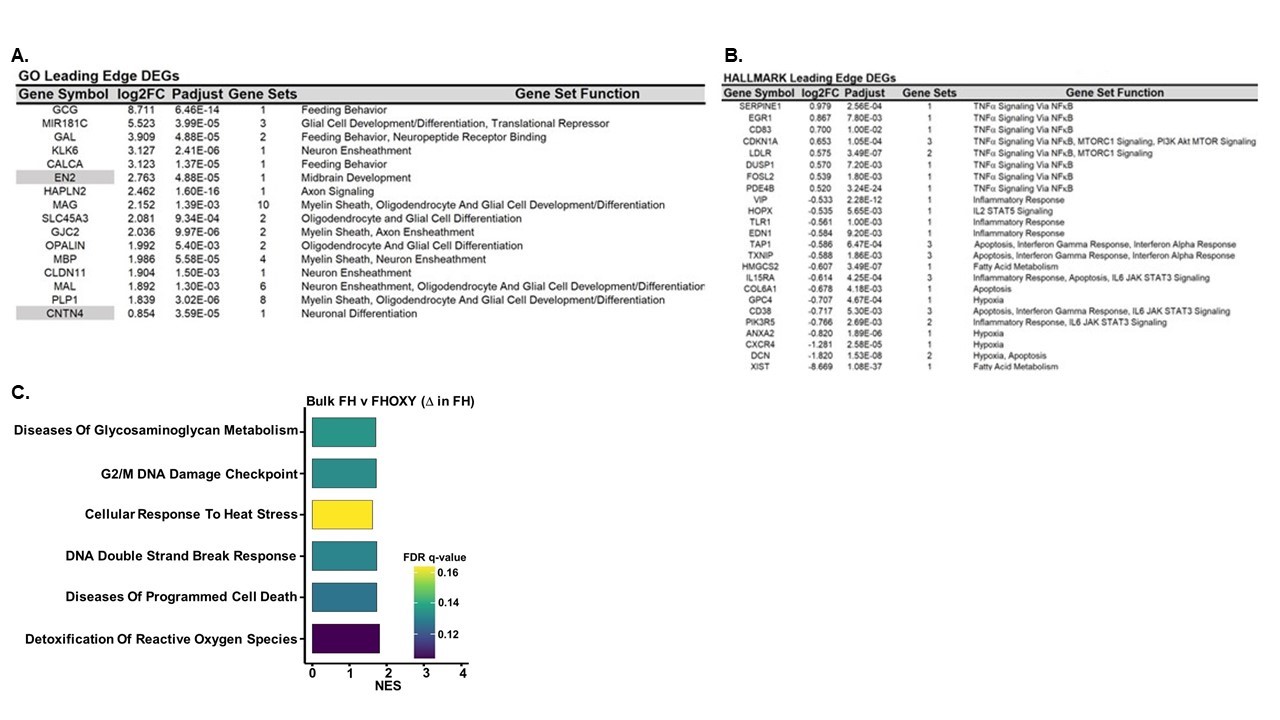

Supplement: Supplementary file 1 — Supplementary Material 1 [file 12993_2024_240_MOESM1_ESM.docx]
